# Vapor‐Assisted Catalysis Enables Precise Construction of MOF‐Derived Hierarchical Carbon Nanoarrays for High‐Performance Supercapacitors

**DOI:** 10.1002/advs.202524363

**Published:** 2026-01-11

**Authors:** Xiaoyang Deng, Zihao Wan, Huan Xin, Xiaoguang Wang, Wenjuan Wang, Feixiong Chen, Liying Ma, Naiqin Zhao

**Affiliations:** ^1^ Laboratory of Advanced Materials and Energy Electrochemistry Institute of New Carbon Materials College of Materials Science and Engineering Taiyuan University of Technology Taiyuan Shanxi China; ^2^ College of Chemistry and Chemical Engineering Taiyuan University of Technology Taiyuan Shanxi China; ^3^ School of Materials Science and Engineering Tianjin University Tianjin China; ^4^ Faculty of Biochemistry and Molecular Medicine Disease Networks Research Unit University of Oulu Oulu Finland

**Keywords:** carbon nanotubes, hierarchical carbon arrays, metal‐organic frameworks, supercapacitor

## Abstract

The synthesis of MOF‐derived carbons with high surface area and conductivity is challenged by a fundamental trade‐off between architectural preservation and catalytic graphitization. Here, we introduce a spatially decoupled, vapor‐assisted pyrolysis strategy where a Co‐MOF precursor array is physically separated from a vapor‐generating Zn‐MOF auxiliary. During pyrolysis, a remote Zn vapor flux simultaneously preserves the precursor's nanosheet morphology by suppressing Co nanoparticle aggregation and catalytically grows dense carbon nanotube (CNT) arrays. This process yields an integrated 0D–1D–2D hierarchical carbon architecture with high surface area and graphitization. As a self‐supporting supercapacitor electrode, the optimized material delivers a specific capacitance of 360 F g^−1^, excellent rate capability (53% retention at 50 A g^−1^), and robust cycling stability. Mechanistic studies and density functional theory calculations confirm the pivotal role of Zn vapor in modulating Co catalysis for morphology control and reveal a synergy between heteroatoms and cobalt that optimizes K^+^ storage kinetics. This remote‐regulation strategy establishes a generalizable platform for designing hierarchical carbon materials for advanced energy storage.

## Graphical abstract

A remote‐control strategy for catalyst design is unveiled for advanced carbon materials. During pyrolysis, zinc vapor from a physically separate source guides cobalt catalysts to construct a hierarchical carbon nanoarray. This approach prevents structural collapse while directing the dense growth of carbon nanotubes, yielding a material with exceptional performance for supercapacitors.

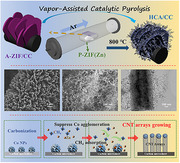

## 1 Introduction

Supercapacitors are pivotal for next‐generation energy storage, offering rapid charge–discharge rates, high power density, and robust cycling stability [1, 2, 3]. The performance of these devices hinges on the carbon‐based electrode architecture, which must simultaneously provide a high specific surface area, hierarchical porosity, and high electrical conductivity [2, 4]. By virtue of their tunable chemistry, well‐defined porosity, and diverse structures, metal–organic frameworks (MOFs) have emerged as exceptional precursors for the synthesis of such multifunctional carbons [4, 5, 6]. However, realizing MOF‐derived carbons that simultaneously exhibit high surface area and conductivity is fundamentally constrained by a trade‐off between promoting graphitization and preserving the often fragile MOF‐derived architectures.

Several strategies have been explored to address this challenge. Reducing atmospheres or liquid metal catalysts can suppress the aggregation of metal nanoparticles and promote the growth of conductive carbon nanotube (CNT) networks [7, 8, 9], but these methods are limited by safety concerns, high costs, and operational complexity. Alternatively, solid auxiliaries such as melamine can enhance CNT formation and partially support the framework; [10, 11, 12] however, mismatches between their thermal decomposition profiles and those of the MOF precursors often lead to structural degradation and/or imprecise control over CNT growth.

A more common approach involves bimetallic Zn/Co‐zeolitic imidazolate framework (ZIF), in which the in situ volatilization of Zn provides spatial confinement for the Co nanoparticles [6, 13, 14]. This strategy, however, is constrained by an inherent stoichiometric dilemma. Optimizing the Zn/Co ratio for morphology preservation inevitably alters the concentration of the catalytic metal (Co). This coupling leads to a suboptimal compromise: low Co content results in poor CNT density and graphitization, whereas high Co content causes catalyst aggregation, negating the role of Zn. Furthermore, this premixed configuration makes it methodologically challenging to decouple the effects of catalyst dilution from the spatial confinement provided by Zn vapor. This unresolved trade‐off has impeded the rational design of materials that simultaneously possess a high surface area and a dense, highly conductive CNT network. Consequently, a strategy that spatially and functionally decouples the architectural preservation agent from the catalytic precursor is highly desirable yet has remained underdeveloped.

Unlike conventional premixed strategies in which the structural spacer (Zn) and the catalyst (Co) are chemically coupled and spatially indistinguishable, our approach introduces a spatially decoupled, vapor‐assisted catalytic pyrolysis strategy. This distinct separation allows the Zn vapor flux to be tuned independently of the Co precursor, breaking the stoichiometric constraints. In this approach, a physically separate ZIF‐8 powder (P‐ZIF(Zn)) serves as a remote vapor source, whose thermal evolution is precisely synchronized with the pyrolysis of the primary Co‐MOF precursor. The resulting flux of zinc vapor and reducing gases acts as a tandem regulator, modulating Co catalyst activity to suppress agglomeration. This controlled catalysis simultaneously preserves the precursor's 2D microsheet architecture and directs the high‐density growth of CNTs, yielding an integrated 0D (carbon nano‐onions)–1D (CNTs)–2D (microsheet) hierarchical carbon architecture (HCA). Mechanistic studies and density functional theory (DFT) calculations reveal the pivotal role of zinc vapor in dynamically regulating Co agglomeration and facilitating a highly conductive network. Concurrently, optimized Co/N/O doping further enhances ion‐storage kinetics by fine‐tuning K^+^ adsorption energies. Consequently, the resulting HCA, when employed as a binder‐free supercapacitor electrode, demonstrates high specific capacitance, superior rate capability, and exceptional cycling stability. This remote‐regulation strategy is generalizable, establishing a versatile platform for the rational design of advanced hierarchical carbon materials.

## 2 Results and Discussion

**FIGURE 1:**
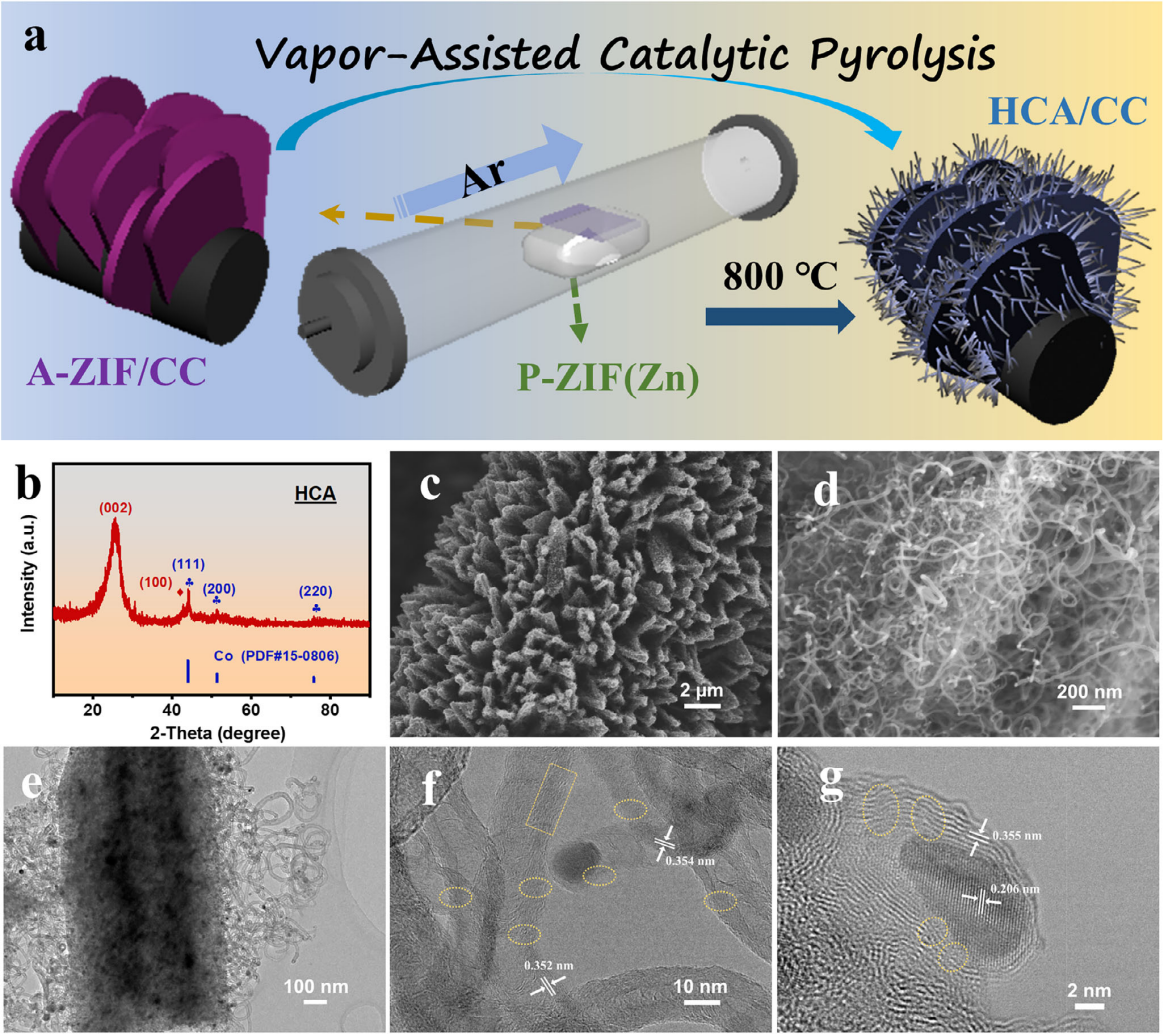
(a) Schematic illustration of the construction of HCA via a facile gas‐phase‐confined catalysis process. (b) XRD pattern of HCA800; (c, d) SEM images, (e) TEM image, and (f, g) HRTEM images of HCA800.

The synthesis strategy for the hierarchical porous carbon nanoarray material is schematically illustrated in Figure 1a. Initially, a cobalt‐containing MOF microsheet array (A‐ZIF) was grown directly on a flexible carbon cloth (CC) substrate. Subsequently, for the vapor‐assisted catalytic pyrolysis, the A‐ZIF‐coated CC was placed in a tube furnace adjacent to but physically separate from the P‐ZIF(Zn). Upon pyrolysis, P‐ZIF(Zn) decomposed, releasing a vapor stream of zinc and reducing gases that enveloped the A‐ZIF precursor. This vapor‐phase regulation suppressed Co agglomeration to preserve the integrated morphology and maintained the high catalytic activity of metallic cobalt for the subsequent growth of CNT arrays. Through this vapor‐assisted catalytic pyrolysis process, the A‐ZIF was transformed into a 2D carbon microsheet array, featuring dense CNT arrays grown on each side, forming the HCA material after acid etching to remove excess metal. The X‐ray diffraction (XRD) patterns in Figure S1 confirm the successful fabrication of both the P‐ZIF(Zn) powder and the A‐ZIF arrays on CC [15, 16, 17]. The XRD pattern of the final product obtained at 800°C, HCA800, (Figure 1b) exhibits two prominent diffraction peaks at 26° and 43°, corresponding to the (002) and (100) crystal planes of graphite carbon, respectively [18, 19]. Their sharpness indicates a high degree of graphitization in HCA800, which is crucial for electrical conductivity. Additionally, three diffraction peaks corresponding to metallic cobalt (PDF#15‐0806) are observed, confirming the presence of Co nanoparticles embedded within the carbon matrix [7, 14].

Scanning electron microscopy (SEM) reveals a uniform 2D microsheet array grown on the carbon cloth scaffold (Figure 1c). Crucially, this architecture was preserved during pyrolysis without structural collapse (Figure S2c,d). Magnified SEM images (Figure 1d; Figure S3) reveal that the microsheet surfaces are covered with densely interwoven CNTs, forming a distinctive “array‐on‐array” architecture. The CNTs have an average diameter of 18.91 nm (Figure S4). Bright white spots at the tips of the CNTs are identified as cobalt nanoparticles that remained after acid etching. Transmission electron microscopy (TEM) of an individual microsheet confirms the dense and uniform coverage of intertwined CNTs (Figure 1e), consistent with the SEM observations. High‐resolution TEM (HRTEM) reveals the multi‐walled nature of the CNTs, with a graphitic interlayer spacing of ∼0.35 nm (Figure 1f). The presence of encapsulated catalyst particles at the CNT tips indicates a tip‐growth mechanism [7, 20]. These graphitic layers are rich in defects, such as discontinuous and fragmented planes, which are expected to create additional active sites for ion storage. Similarly, defect‐rich carbon nano‐onions (CNOs) are observed, which are catalyzed by Co nanoparticles exposing their (111) facets (d‐spacing = 0.206 nm) (Figure 1g) [21, 22]. Taken together, this multi‐scale characterization confirms that HCA800 is a fully integrated 0D–1D–2D hierarchical material, comprising carbon nano‐onions, CNT arrays, and microsheet arrays. This integrated 0D–1D–2D carbon material synergistically combines multi‐dimensional components to afford multi‐scale transport advantages. Specifically, the 2D microsheet backbone preserves the original MOF‐derived architecture, providing a continuous, mechanically robust scaffold and primary ion‐accessible surface. The 1D CNT network acts as conductive highways for rapid electron transport along the axial direction, interconnecting the 2D sheets to create a 3D conductive network that bridges adjacent microsheets and facilitates 3D charge transport. Meanwhile, the 0D carbon nano‐onions provide additional defect‐rich active sites for ion storage and contribute to the hierarchical porosity. Collectively, this multi‐dimensional integration enables fast in‐plane electron conduction, efficient cross‐plane CNT wiring, and abundant accessible surface/defect sites, thereby optimizing both charge transport and electrochemical activity.

**FIGURE 2:**
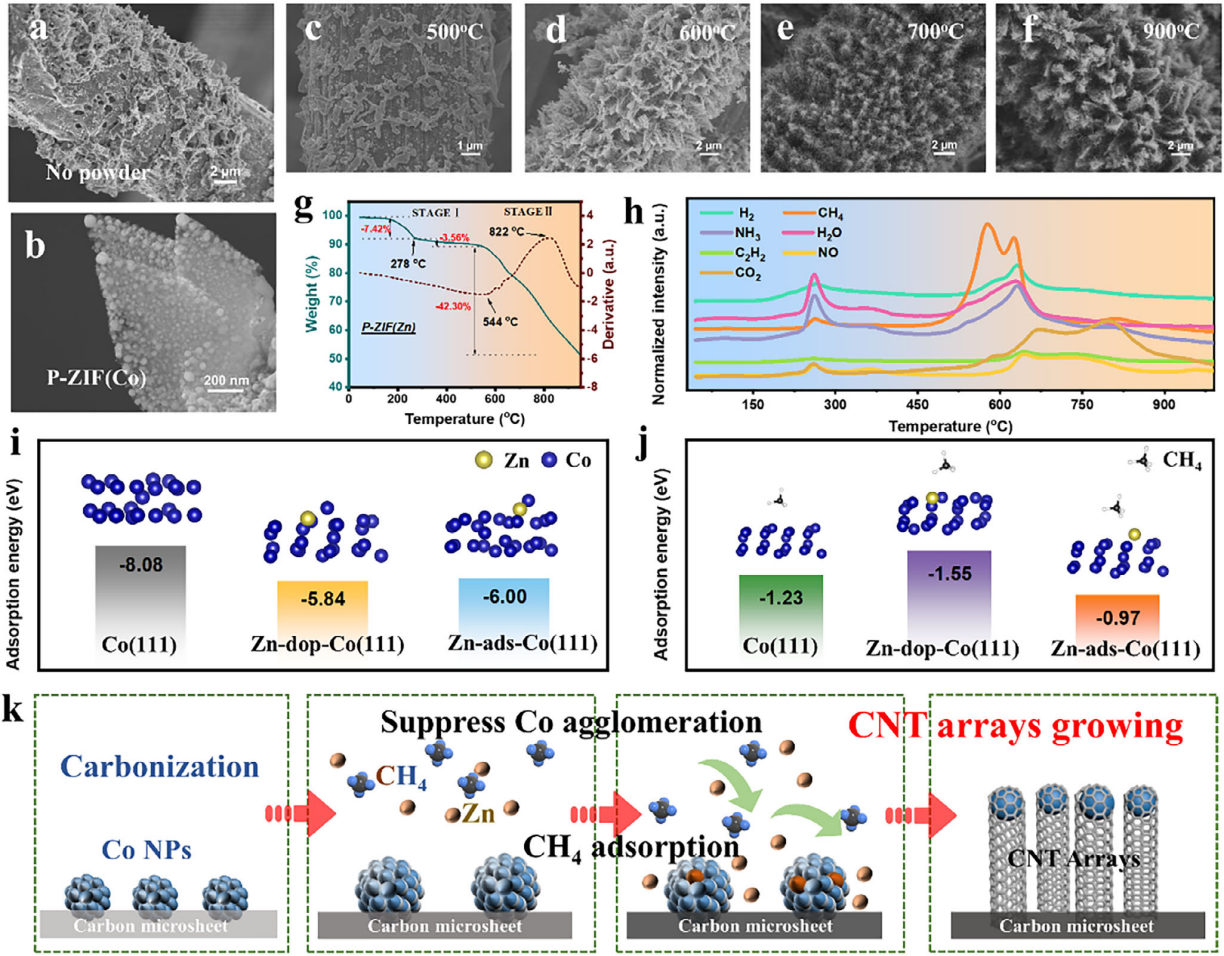
(a) SEM image of the carbon material obtained through direct carbonization of A‐ZIF without ZIF powder; (b) SEM image of SCA with P‐ZIF(Co); (c–f) SEM images of HCA materials obtained at different temperatures; (g) TG/DSC and (h) TG‐MS curves of the P‐ZIF(Zn); (i) Co and (j) CH_4_ adsorption energy on different models; (k) Schematic diagram of the formation process for CNT arrays.

To elucidate the formation mechanism of the hierarchical architecture, we systematically modulated the synthesis parameters. A series of control experiments first established the critical role of P‐ZIF(Zn) as a remote vapor source. Direct pyrolysis of the A‐ZIF precursor at 800°C in the absence of P‐ZIF(Zn) resulted in the complete collapse of the microsheet array. The carbon fiber surface became covered with large metallic agglomerates and showed evidence of severe etching (Figure 2a; Figure S5). This baseline experiment confirms that an external regulatory agent is essential to suppress Co agglomeration and preserve the precursor morphology during carbonization.

Having established this necessity, we sought to identify the key active components. To isolate the function of the auxiliary agent, P‐ZIF(Zn) was compared with its analogues P‐ZIF(Co) and P‐ZIF(ZnCo) (Figure S6). Compared with the HCA800 without acid‐etching (Figure S7), the P‐ZIF(Co) vapor source led to severe Co agglomeration and degradation of the microsheet architecture (Figure 2b; Figure S8). In contrast, P‐ZIF(ZnCo) effectively suppressed metal agglomeration and preserved the precursor morphology, even promoting the growth of nascent CNTs (Figure S9). These results directly implicate zinc as the crucial element for both structural preservation and CNT nucleation. We next investigated whether the delivery method of zinc was critical. Substituting P‐ZIF(Zn) with conventional zinc sources, such as nano‐Zn or nano‐ZnO powders, failed to protect the structure, leading to collapse and etching (Figure S10). Crucially, even when nano‐Zn powder was combined with a separate gas source (P‐ZIF(Co)), the synthesis still failed to produce CNTs, yielding a product identical to that from P‐ZIF(Co) alone (Figure S11). Furthermore, melamine, a common N‐rich auxiliary agent, also failed to maintain the microsheet array (Figure S12). These control experiments collectively demonstrate that neither zinc vapor alone nor a generic protective atmosphere is sufficient. The superior performance is unique to the MOF‐derived zinc vapor source.

Based on these findings, we propose a dual‐action mechanism enabled by the P‐ZIF(Zn) framework. Direct visual evidence for this mechanism is provided by the TEM images of the unetched HCA800 (Figure S7). Unlike the large agglomerates observed in control samples without vapor regulation, the unetched HCA800 reveals uniformly dispersed, small Co nanoparticles embedded within the matrix, confirming that the Zn vapor effectively suppresses sintering during the critical nucleation phase. We attribute its efficacy to the atomic‐level dispersion of Zn ions within the ZIF structure. Upon pyrolysis, this arrangement facilitates a synchronized and sustained release of (i) reducing gases that form a protective atmosphere to prevent microsheet collapse, and (ii) a controlled flux of zinc vapor. This fine, homogeneous Zn vapor, likely composed of nanoclusters or atoms, is distinct from the coarse vapor generated from bulk sources, which requires higher temperatures. This controlled flux is essential for templating the delicate precursor architecture and catalyzing CNT growth. The MOF framework thus transforms zinc from a simple additive into a strategic component that enables high‐precision synthesis of the hierarchical architecture.

The transient, physical role of zinc was then confirmed. Energy‐dispersive X‐ray spectroscopy (EDS) mapping of the unetched HCA800 revealed a uniform distribution of residual Zn alongside C, N, O, and Co (Figure S13). X‐ray photoelectron spectroscopy (XPS) confirmed that this zinc exists in a purely metallic state (Zn^0^) (Figure S14). This finding confirms that zinc functions as a dynamic physical mediator during carbonization rather than as a permanent chemical dopant. The dosage of P‐ZIF(Zn) was also found to be critical, with an optimal window governing both CNT yield and morphology (Figures S15 and S16), underscoring the controllability of the process.

Finally, the morphological evolution as a function of temperature was mapped from 500 to 900°C, yielding samples designated HCA500, HCA600, HCA700, and HCA900, respectively. The microsheet array disintegrated at 500°C but was preserved above 600°C, albeit with large Co particles (Figure 2c,d; Figure S17a,b). A dense CNT network emerged on the microsheet at 700°C (∼26 nm diameter) (Figure 2e; Figure S4a,c), with optimal density and morphology achieved at 800°C. At 900°C, the CNT density increased, but their length was reduced (diameter ∼21 nm) (Figure 2f; Figure S4c and S17d). This systematic study reveals a delicate thermal balance. Temperature dictates the preservation of the primary microsheet scaffold and simultaneously regulates the secondary growth of CNTs by controlling the size and activity of the Co catalyst particles.

To elucidate the functional dynamics of the auxiliary agent, the thermal decomposition of P‐ZIF(Zn) was investigated. Thermogravimetric analysis (TGA) and mass spectrometry (TG‐MS) revealed a multi‐step process (Figure 2g,h). Following solvent removal below 278°C, the ZIF framework begins to decompose, with vigorous ligand fragmentation occurring between 550–650°C. Consistent with the pyrolysis of ZIF‐8, this process is proposed to involve the initial formation of ZnO intermediates [23, 24, 25], which are subsequently reduced to volatile metallic zinc at higher temperatures [26]. A critical finding emerged from comparing the thermal profiles of the main precursor (A‐ZIF) and the auxiliaries (P‐ZIF(Zn), P‐ZIF(Co)). Their initial decomposition stages are virtually synchronous (Figure S18) [27]. This temporal alignment is crucial, as it ensures the protective gases and regulatory zinc vapor are released precisely when the primary A‐ZIF structure becomes vulnerable.

Density Functional Theory (DFT) calculations provided theoretical validation for zinc's pivotal role. The effect of zinc vapor on Co agglomeration was modeled on Co(111) surfaces with either doped (Zn‐dop‐Co(111)) or adsorbed zinc atoms (Zn‐ads‐Co(111)). The calculations showed that in both scenarios (Figure 2i), the presence of zinc significantly weakens the binding energy of incoming cobalt atoms, substantiating its function as a potent anti‐agglomeration agent. Furthermore, the role of zinc in governing the catalytic activity of cobalt nanoparticles was investigated by calculating the methane adsorption energy, a key descriptor of C─H bond activation and a widely used indicator for the initial step of CNT growth [28]. The calculations of methane adsorption energy revealed a dual effect of zinc on cobalt's catalytic activity: low concentrations (doping) enhance activity, whereas high concentrations (surface adsorption) inhibit it (Figure 2j). This dual regulatory function explains our experimental observation at 900°C, where an increased rate of zinc volatilization leads to higher surface coverage on Co particles, thereby inhibiting CNT growth and resulting in shorter nanotubes.

Synthesizing these experimental and theoretical insights, we propose a comprehensive mechanism for the vapor‐assisted catalytic pyrolysis (Figure 2k). The process initiates with the synchronous decomposition of P‐ZIF(Zn) and A‐ZIF. The pyrolysis of P‐ZIF(Zn) generates two crucial outputs: a flux of reducing gases and intermediate ZnO/Zn species confined within a porous carbon matrix. This confinement is hypothesized to facilitate a more efficient carbothermal reduction, in which the local temperature is higher than the bulk boiling point of zinc, thereby generating a sustained, finely dispersed zinc vapor flux [29, 30]. This results in synergistic vapor‐phase regulation. The gas flux creates a reducing atmosphere that protects the microsheet scaffold from collapse and prevents oxidation of the Co catalysts. Concurrently, the zinc vapor acts as a dynamic physical spacer to prevent Co sintering. This precise control maintains a high density of small, active cobalt nanoparticles, which catalyze the growth of CNT arrays. Simultaneously, some of these stabilized Co nanoparticles catalyze the formation of CNOs, completing the final 0D‐1D‐2D hierarchical architecture.

**FIGURE 3:**
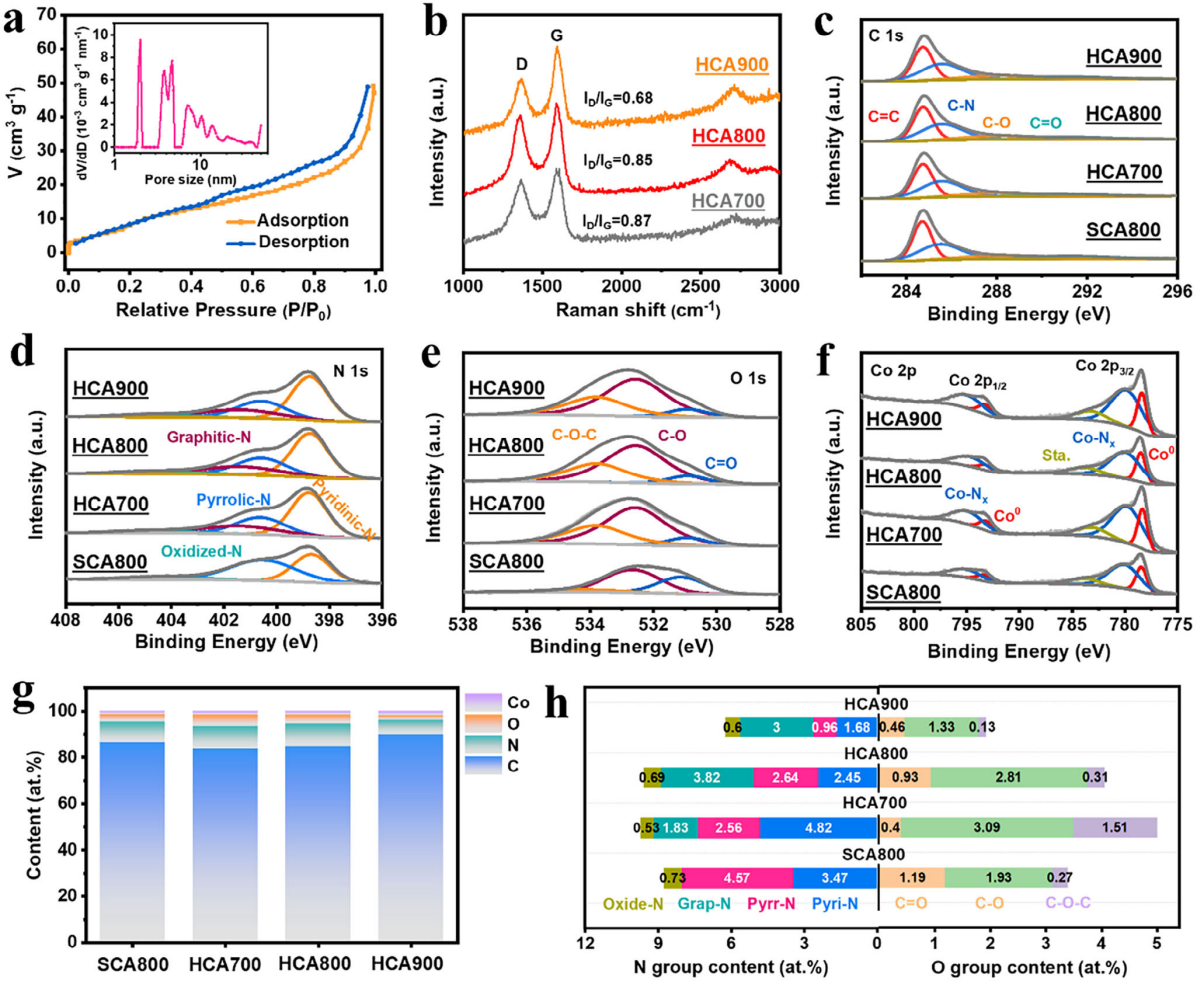
(a) Nitrogen adsorption/desorption isotherm of HCA800; inset shows the pore size distribution. (b) Raman spectra of HCAs. High‐resolution XPS spectra for (c) C 1s, (d) N 1s, (e) O 1s, and (f) Co 2p spectra of HCAs and SCA. (g) Total elements content and (h) contents comparison of different N and O species derived from XPS spectra.

The porous architecture of HCA800 was characterized by nitrogen adsorption/desorption measurements. The isotherm of HCA800 (Figure 3a) exhibits a pronounced hysteresis loop in the P/P_0_ range of 0.4–1.0, indicating a hierarchical pore structure that is rich in mesopores and macropores. After normalizing for the carbon cloth substrate, the active material's intrinsic BET specific surface area was estimated to be approximately 549 m^2^ g^−1^. The pore size distribution confirms a broad range of pores (inset, Figure 3a) originating from the A‐ZIF precursor template, etching effects of Co and Zn vapor, and the interstices between CNTs and CNOs.

Raman spectroscopy was used to evaluate the degree of graphitization as a function of synthesis temperature (Figure 3b). All HCA samples displayed the characteristic D‐band (∼1350 cm^−1^) and G‐band (∼1580 cm^−1^). The low intensity ratios of I_D_/I_G_ confirm that the vapor‐regulated cobalt catalyst effectively promoted graphitization. Notably, the I_D_/I_G_ ratio systematically decreases with increasing temperature (from HCA700 to HCA900), signifying enhanced graphitic crystallinity, which is advantageous for rapid electron transport.

XPS was employed to investigate the surface chemistry. Survey scans confirmed that all HCA samples and the control SCA800 (synthesized without P‐ZIF(Zn)) are composed of C, N, O, and Co (Figure 3g; Figure S19 and Table S1). For instance, HCA800 contains 9.60 at% N, 4.05 at% O, and 1.50 at% Co. High‐resolution C 1s spectra revealed C═C (284.4 eV), C─N (285.2 eV), C─O (287.1 eV), and ─C═O (290.1 eV) species [31, 32], confirming successful heteroatom incorporation (Figure 3c). The total N and O content in the HCA series decreased with increasing temperature, consistent with their improved crystallinity observed in Raman spectra. Crucially, a comparison between HCA800 and SCA800 reveals that the vapor‐assisted process profoundly governs the final surface chemistry. This regulation does not arise from a direct chemical interaction but is instead an indirect consequence. The Zn vapor‐regulated pathway, by promoting a more ordered and graphitized structure, fundamentally alters the types and proportions of N and O species that can be thermodynamically stabilized on the carbon surface at high temperatures.

Analysis of the N 1s spectra provides deeper insight into this regulation. The spectra were deconvoluted into pyridinic‐N (398.3 eV), pyrrolic‐N (399.9 eV), graphitic‐N (401.0 eV), and oxidized‐N (402.0 eV) (Figure 3d) [33, 34]. A striking contrast emerges from the quantitative analysis (Figure 3h): SCA800 is rich in pyridinic/pyrrolic‐N but contains negligible graphitic‐N, whereas all HCA samples possess a significant proportion of the latter. Within the HCA series, the fraction of graphitic‐N increases with temperature. This finding provides compelling evidence for our proposed indirect regulation mechanism. The highly ordered graphitic framework in the HCA samples, a direct result of the vapor‐assisted catalysis as confirmed by Raman spectroscopy (Figure 3b), provides a thermodynamically favorable lattice for incorporating thermally stable graphitic‐N. Conversely, the amorphous and defect‐rich structure of SCA800 preferentially retains less stable pyridinic and pyrrolic‐N configurations at edge sites. Concurrently, the abundant edge defects of the CNTs serve as trapping sites for pyridinic‐N. HCA800 thus achieves a synergistic balance of graphitic‐N (enhancing conductivity) and pyridinic‐N (providing active sites and improving wettability).

The vapor‐assisted process also regulates the oxygen and cobalt species. The O 1s spectrum (Figure 3e) similarly shows multiple functionalities including C═O (530.8 eV), C─O/N─O (532.5 eV), and C─O─C (534.7 eV) [35]. Notably, the distribution of these oxygen species also depends on the synthetic route (Figure 3h). The HCA samples exhibit a higher relative content of C─O functionalities compared to the unregulated SCA800. This does not imply a direct promotional effect from the auxiliary, but rather reflects how the different carbonization pathways result in distinct surface terminations and defect structures, which in turn stabilize different oxygen‐containing groups. Despite acid etching, trace amounts of cobalt persist. The Co 2p spectra (Figure 3f) confirm the dual existence of both metallic Co (778.6 eV) and Co─N moieties (780.8 eV) [22, 36]. The residual cobalt is advantageous as it can modulate the interfacial microenvironment, optimize electrochemical adsorption/desorption behavior, and facilitate electron transfer, leading to superior overall electrochemical performance.

**FIGURE 4:**
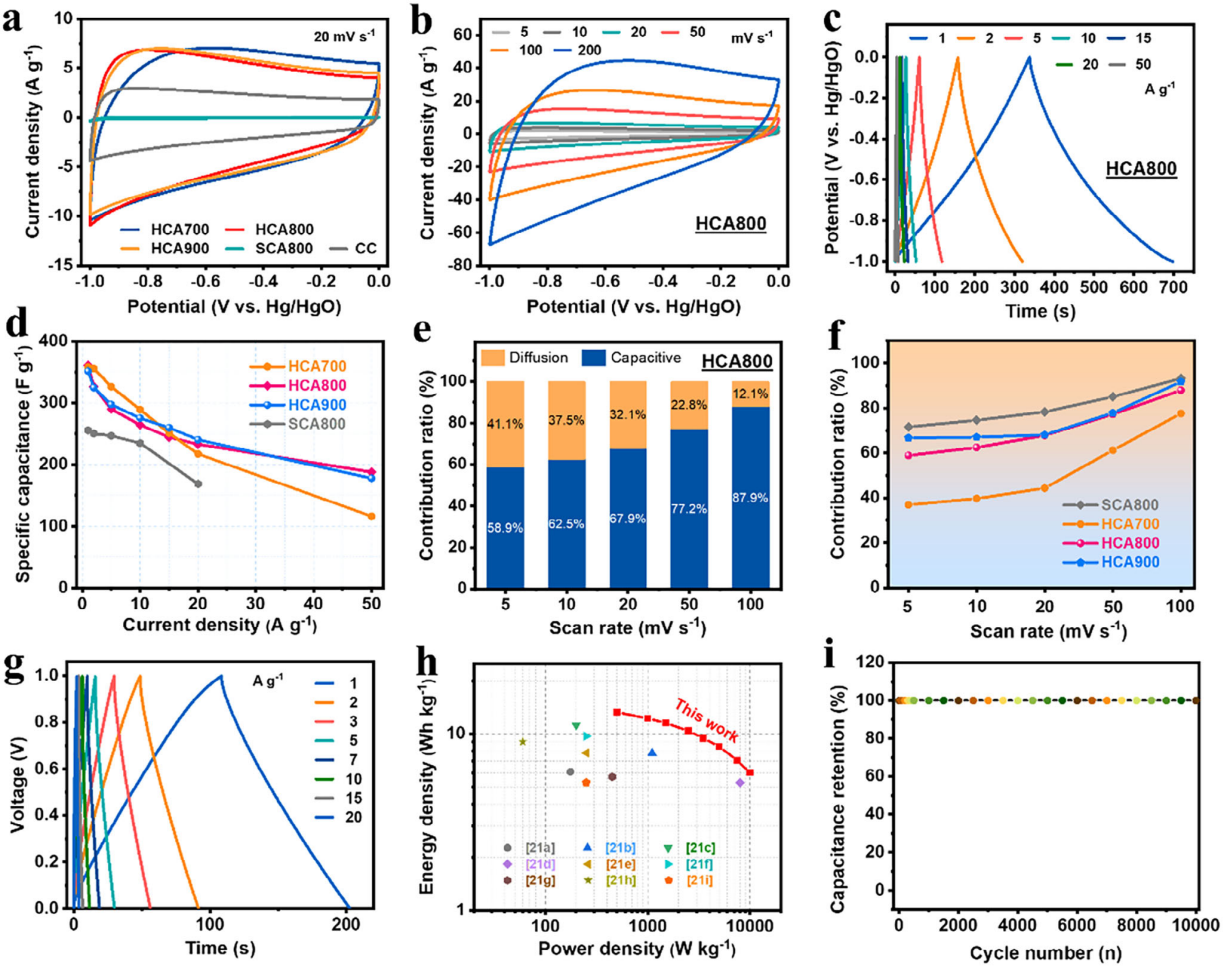
(a) CV curves of all the electrodes at 20 mV s^−1^; (b) CV curves of the HCA800 electrode at different scan rates; (c) GCD curves of the HCA800 electrode; (d) Specific capacitance comparison of HCAs and SCA800 electrodes; (e) Capacitive contribution of the HCA800 electrode at various scan rates; (f) Comparison of capacitive contribution; (g) GCD profiles, (h) Ragone plot, and (i) cycling performance curves of the the HCA800‐based symmetrical supercapacitor.

The electrochemical performance was first evaluated in a three‐electrode configuration using a 6 m KOH electrolyte. Cyclic voltammetry (CV) curves of the HCA series and the control sample SCA800 were compared (Figure 4a). All HCA electrodes exhibited quasi‐rectangular shapes, confirming a predominantly electric double‐layer capacitive (EDLC) storage mechanism [2]. Notably, the CV profiles of HCA800 and HCA900 were significantly more rectangular than that of HCA700, indicating superior conductivity and electrochemical kinetics. In contrast, the smaller CV area of SCA800 suggested a lower specific capacitance, attributed to its less developed hierarchical structure and fewer accessible active sites. The negligible current response from bare CC confirms its insignificant capacitance contribution (Figure S20).

The rate capability of HCA800 was exceptional. It maintained a well‐defined rectangular CV shape even at a high scan rate of 200 mV s^−1^ (Figure 4b), and its galvanostatic charge–discharge (GCD) profiles remained highly symmetric and nearly linear across a wide range of current densities (Figure 4c; Figure S21) [2]. This high‐rate response contrasts sharply with the performance of HCA700, whose distorted profiles reflect kinetic limitations from its lower degree of graphitization. Quantitatively, HCA800 delivered a high specific capacitance of 360 F g^−1^ at 1 A g^−1^ and retained an impressive 191 F g^−1^ (53% retention) at a demanding current density of 50 A g^−1^ (Figure 4d). This rate performance decisively surpasses that of both SCA800 and HCA700, validating the vapor‐assisted catalytic strategy. The performance of HCA800 is also highly competitive with that of recently reported carbon‐based materials (Tables S2 and S3).

To elucidate the origin of this high‐rate capability, a kinetic analysis using Dunn's method was performed (Figure 4e,f) [3, 37]. The analysis revealed that capacitive‐controlled processes dominate the charge storage in HCA800, particularly at high scan rates. This confirms that its superior performance arises from an optimized combination of a hierarchical architecture, which ensures rapid ion access, and high electrical conductivity, which facilitates efficient electron transport. While SCA800 also showed a high capacitive contribution, its simpler structure limited its absolute capacity. Conversely, the high heteroatom content in HCA700 induced pseudocapacitance, but its poor conductivity created a kinetic bottleneck, limiting its power characteristics.

To assess its practical applicability, a symmetric supercapacitor was fabricated using HCA800 electrodes. The device operated within a stable 0–1.0 V window, exhibiting quasi‐rectangular CV curves and symmetric, triangular GCD profiles even at 200 mV s^−1^, indicative of ideal capacitive behavior and high‐rate reversibility (Figure 4g; Figure S22). This translated to a highly competitive energy‐power profile. At 1 A g^−1^, the symmetric supercapacitor delivers a substantial specific capacitance of 94.20 F g^−1^ (Figure S23). Furthermore, the corresponding Ragone plot (Figure 4h) highlights its competitive performance, achieving a high energy density of 13.08 Wh kg^−1^ at a power density of 500 W kg^−1^ and reaching a peak power density of 10 kW kg^−1^. Crucially, these values are superior or highly competitive relative to those of recently reported carbon‐electrode‐based supercapacitors [38, 39, 40, 41, 42, 43, 44, 45, 46]. This performance stems from superb charge transfer and ion diffusion kinetics, as revealed by electrochemical impedance spectroscopy (EIS) plots (Figure S24). The small high‐frequency semicircle signifies low charge transfer resistance (R_ct_), while the nearly vertical line in the low‐frequency region indicates efficient ion diffusion pathways [47]. Together, these features confirm rapid mass transport and charge transfer kinetics within the symmetric supercapacitor.

Crucially, the device exhibited outstanding long‐term durability. It retained 100% of its initial capacitance over 10,000 charge–discharge cycles at a high current density of 10 A g^−1^ (Figure 4i). Post‐cycling SEM analysis confirmed that the intricate hierarchical structure remained fully intact (Figure S25), while XPS analysis revealed no discernible change in either the elemental composition or the chemical states of the constituting elements in the HCA800 electrode after prolonged cycling (Figure S26). This exceptional structural and chemical robustness provides compelling evidence that the vapor‐assisted strategy enables materials that combine high electrochemical performance with ultra‐stable operation.

**FIGURE 5:**
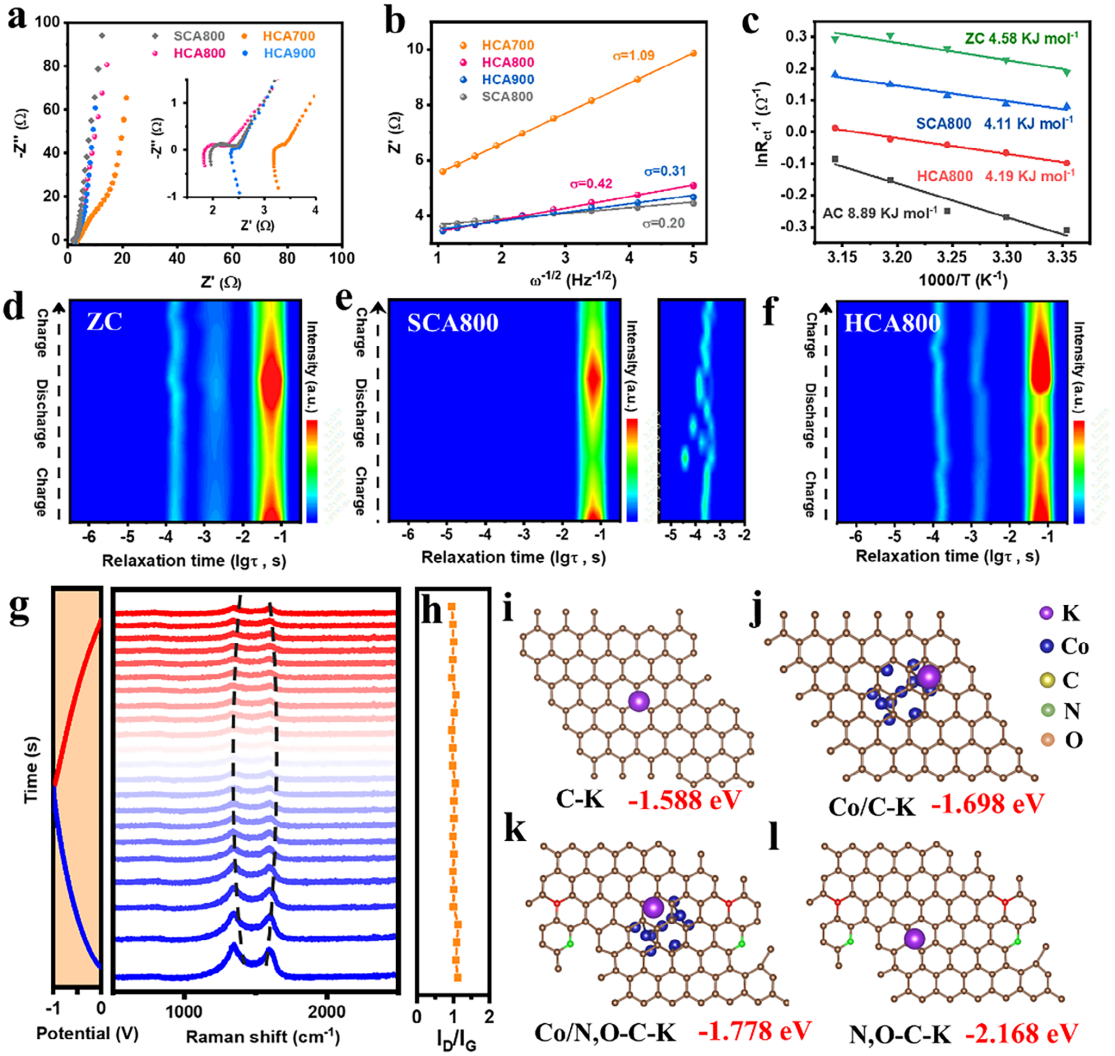
(a) Nyquist plots of the HCAs and SCA800 electrodes; (b) Linear fit for the relationship between Z′ and *ω* ^−1/2^. (c) Activation energy for the AC, ZC, SCA800 and HCA800 electrodes according to the temperature‐dependent EIS; (d–f) Contour maps of DRT results obtained from the in situ EIS during the charging/discharging process; (g) GCD curve and in situ Raman spectra of the HCA800 electrode; (h) Corresponding ratios of I_D_/I_G_; (i–l) Adsorption energy of K^+^ ion on different carbon models.

Electrochemical impedance spectroscopy (EIS) was used to probe the ion diffusion and charge transfer dynamics. Nyquist plots revealed that HCA900 possesses the lowest Rct, followed by HCA800 and HCA700; this trend correlates directly with their increasing degree of graphitization (Figure 5a). Ion diffusion kinetics were quantified by calculating the Warburg coefficient (σ) from the slope of *Z’* versus *ω* ^−^ *^0.5^* plots (Figure 5b) [48]. Because the diffusion coefficient is inversely proportional to σ, a smaller slope indicates faster diffusion kinetics. HCA700 exhibited the highest σ value (lowest diffusion coefficient), a result attributed to its high concentration of heteroatoms (Co, N, O) and lower graphitization. While heteroatom doping can provide pseudocapacitive sites, an excessive concentration can create strong adsorption sites that hinder K^+^ ion diffusion. An illustrative comparison arises between HCA800 and HCA900: despite HCA900 having more Co, the higher N/O content of HCA800 results in a slightly larger σ, suggesting that, beyond an optimal level, strong K^+^ interactions with N/O sites may impose a more significant kinetic barrier than the presence of well‐dispersed Co. Notably, the control sample SCA800 exhibited the fastest ion diffusion (lowest σ). However, its overall electrochemical performance remains poor due to its lower specific surface area and lack of a robust CNT network. This result conclusively demonstrates that while fast diffusion is important, it is the synergistic interplay between a hierarchical morphology (for efficient pathways and active sites) and an optimized level of heteroatom doping (for balanced adsorption/desorption kinetics) that dictates superior supercapacitor performance.

To isolate the contributions of different chemical components to the charge transfer kinetics, the activation energy (Ea) was determined from temperature‐dependent EIS and the Arrhenius equation [15, 49]. Activated carbon (AC), cobalt‐free ZIF‐8‐derived carbon powder (ZC), and SCA800 electrodes were employed as control samples to deconstruct the contributions of different components. As shown in Figure 5c, HCA800 exhibited a low activation energy of 4.19 kJ mol^−1^, a value comparable to that of SCA800 but significantly lower than those of ZC and AC, signifying a substantially reduced interfacial charge‐transfer barrier. A comparative analysis provides clear insights: the lower *Ea* of the nitrogen‐rich ZC relative to AC confirms the beneficial role of N doping in facilitating charge transfer. More importantly, the superior performance of the cobalt‐containing HCA800 over the N‐doped ZC underscores the dual functionality of cobalt, which not only catalyzes graphitization to enhance intrinsic conductivity but whose residual metallic species also serve as active sites that accelerate electron transfer.

To unravel the kinetic origins of the excellent electrochemical performance of the HCA800 electrode, in situ EIS coupled with Distribution of Relaxation Times (DRT) analysis was employed to monitor its dynamic evolution during cycling (Figure 5d–f). The DRT analysis deconvolves the electrochemical impedance into distinct processes occurring at different time constants. [50, 51, 52, 53] In the low‐frequency domain (τ ≈ 10^−2^–10^−1^ s), corresponding to capacitive processes, the subtle shifts and broadening observed in HCA800 are interpreted not as instability, but as a manifestation of its complex electrochemistry arising from a spectrum of charge storage sites (EDLC, pseudocapacitance at N/O and Co‐N_x_ sites). Critically, the overall peak profile remains remarkably stable and reversible throughout the cycle, indicating that these diverse charge storage pathways are kinetically efficient. The most critical insights emerge from the mid‐frequency region (τ ≈ 10^−3^–10^−2^ s), which probes the core charge transfer process. HCA800 distinguishes itself with a remarkably stable peak shape, in stark contrast to the significant fluctuations seen in ZC, demonstrating exceptionally robust and efficient interfacial kinetics. In stark contrast, the control samples ZC and SCA800 showed either significant fluctuations (ZC) or a lack of a well‐defined, stable peak (SCA800), directly confirming that the vapor‐regulated hierarchical structure of HCA800 is essential for creating these stable and efficient charge transfer pathways. The high‐frequency domain (τ ≈ 10^−4^–10^−3^ s), associated with surface diffusion and electron transport, solidifies our conclusion. While HCA800 and ZC show stable, linear behavior, the chaotic response of SCA800 highlights a critical flaw: its high cobalt loading, without a robust carbon framework and effective vapor‐mediated regulation, results in poorly dispersed Co particles that disrupt electronic conductivity and interface stability. This comprehensive DRT analysis further confirms the presence of highly efficient and stable charge‐transfer pathways in HCA800.

The structural integrity of HCA800 was further verified by in situ Raman spectroscopy during cycling (Figure 5g). The fully reversible shifts of the D and G bands confirm its exceptional structural resilience, a finding corroborated by post‐cycling SEM imaging (Figure S24). As shown in Figure S27, the 2D band exhibits only subtle variations during charging/discharging, demonstrating the structural robustness of HCA800. Critically, the nearly invariant I_D_/I_G_ ratio not only underscores the absence of irreversible structural degradation, which is the origin of its superior cycling stability, but also points to a predominantly capacitive energy storage mechanism [31, 54].

Furthermore, DFT calculations were performed to theoretically rationalize the synergistic contributions of the heteroatoms and cobalt to K^+^ ion storage kinetics. By systematically modeling the key components—the carbon matrix, N/O dopants, and cobalt nanoparticles—their individual and synergistic roles in interacting with K^+^ ions were isolated [40, 55]. As shown in Figure S28, the density of states (DOS) results reveal that N/O doping and Co incorporation markedly increase and concentrate the electronic states near the Fermi level, enhancing conductivity, electronic coupling, and redox activity. This optimized electronic structure rationalizes the superior rate capability and high specific capacitance of the Co/N,O‐C electrode. The calculations unveil an elegant synergistic mechanism for K^+^ adsorption (Figure 5i–l). The calculations reveal that N,O doping significantly enhances the K^+^ adsorption energy (from −1.588 eV for pristine carbon to −2.168 eV), providing a strong thermodynamic driving force for ion capture. However, excessively strong binding could hinder ion release and slow charge–discharge kinetics. The introduction of nearby cobalt sites moderates this interaction, slightly weakening the binding energy (e.g., to −1.778 eV for Co/N,O─C). This creates a finely tuned “adsorb‐and‐release” landscape in which N,O dopants efficiently capture K^+^ ions, while adjacent Co centers facilitate their rapid and reversible exchange, thereby accelerating overall ion transport and improving rate capability. Furthermore, to probe the electronic contribution to the capacitance, the quantum capacitance was evaluated based on the DOS (details are provided in the Supporting Information). The Co/N,O─C model exhibits the highest quantum capacitance due to the increased carrier density near the Fermi level, which aligns with the experimentally observed superior rate capability. This computationally validated kinetic balance is a direct consequence of the vapor‐assisted catalytic process, which enables simultaneous control over both the hierarchical structure and the intricate surface chemistry.

**FIGURE 6:**
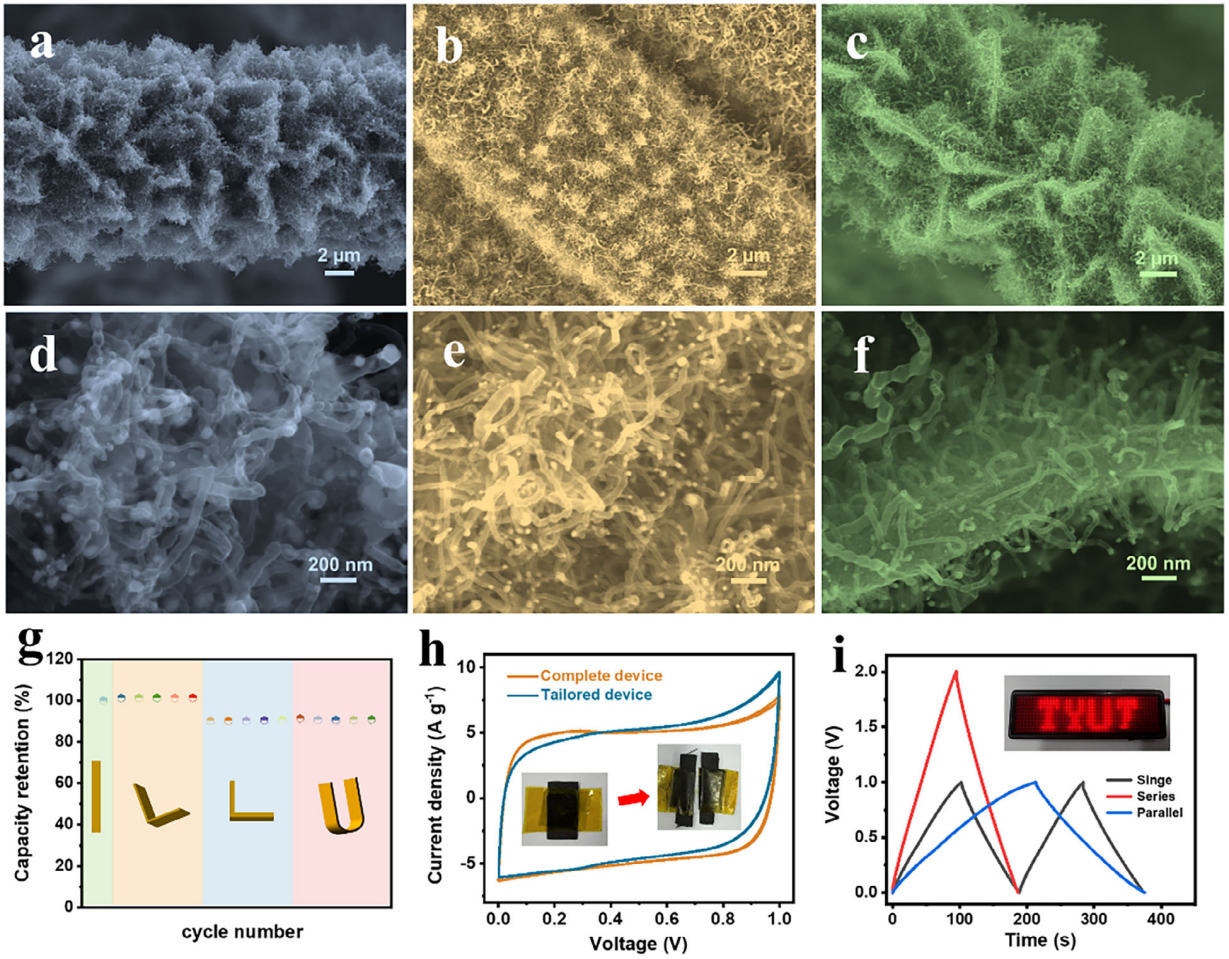
SEM images of (a, d) CoCu‐HCA, (b, e) CoNi‐HCA, and (c, f) CoZn‐HCA. (g) Capacity‐retention ratio of the flexible supercapacitor under various mechanical deformations. (h) CV curves of tailored flexible supercapacitor units. (i) GCD curves for single and two units connected in series and in parallel at 1.0 A g^−1^; inset: illustration of the device powering an LED.

To demonstrate the generalizability of the vapor‐assisted catalytic strategy, it was applied to a range of precursors. First, various bimetallic ZIFs (A‐CoCu‐ZIF, A‐CoNi‐ZIF, and A‐CoZn‐ZIF [16], confirmed by XRD in Figure S29a) were used. Each was successfully converted into a hierarchical carbon array (HCA), although with distinct morphologies that reflect the catalytic nature of the secondary metal (Figure 6a–f). Leveraging the known synergistic catalysis of Co‐Ni alloys, A‐CoNi‐ZIF yielded well‐defined arrays of long CNTs. In contrast, A‐CoCu‐ZIF produced partially collapsed structures, likely due to the different alloying behavior and lower catalytic activity of copper under these conditions. The A‐CoZn‐ZIF precursor produced a lower density of CNTs, suggesting that the Zn already present in the precursor reduces the amount of Co nanoparticles. Despite these morphological differences, XRD patterns (Figure S29b) consistently showed graphitic carbon and metallic Co phases in all samples, confirming the fundamental conversion. The strategy's versatility was further demonstrated by extending it beyond ZIFs to Prussian Blue Analogues (PBAs); Co‐Ni‐PBA was successfully transformed into a uniform, high‐aspect‐ratio CNT array (Figure S30). These results establish the vapor‐assisted method as a robust and versatile platform for engineering hierarchical CNT architectures from a wide range of precursors.

To assess the material's practical viability, a flexible symmetric supercapacitor (FSC) was fabricated using HCA800 electrodes and a PVA/KOH gel electrolyte. As shown in Figures S31–S35, the HCA800‐based FSC exhibits nearly triangular and highly symmetric GCD profiles with high capacitance retention, low self‐discharge, and excellent cycling stability in the gel electrolyte, while the Nyquist/Bode plots and Ragone plot reveal low internal and charge‐transfer resistances together with competitive energy densities over a wide power range, highlighting its robust, fast, and practical solid‐state performance. Furthermore, the device demonstrated excellent mechanical resilience, retaining over 90% of its initial capacitance across various bending angles from 0° to 180° (Figure 6g). The intrinsic flexibility and current‐collector‐free design of the electrode also enabled device customization. Tailored segments, when cut and operated individually, exhibited consistent normalized capacitance and could be reconfigured in series or parallel to achieve tunable voltage and capacitance outputs (Figure 6h,i). As a proof of concept, an integrated device successfully powered a light‐emitting diode (LED) (inset, Figure 6i). Together, these results confirm the material's suitability for high‐performance, adaptable energy storage applications, particularly in wearable electronics.

## 3 Conclusions

In conclusion, we have established a vapor‐assisted catalytic strategy that utilizes in situ generated Zn vapor for the dynamic regulation of catalyst behavior during pyrolysis. This approach enables the synthesis of highly uniform hierarchical carbon arrays (HCAs) by preventing the aggregation of cobalt nanoparticles and preserving the precursor's intricate microsheet morphology during templated carbon nanotube growth. The resulting materials integrate hierarchical porosity with a high degree of graphitization and optimized heteroatom doping, which translates to superior rate capability and long‐term cycling stability when used as supercapacitor electrodes. Mechanistic studies, supported by DFT calculations, reveal two key synergistic effects. First, Zn vapor dynamically modulates the cobalt catalyst's activity, controlling both the graphitization of the carbon framework and the uniform growth of CNTs. Second, the resulting Co/N/O active sites create a finely tuned energy landscape that promotes strong K^+^ adsorption while simultaneously facilitating rapid charge transfer and ion release. This optimized kinetic balance is fundamental to the material's high performance. The generalizability of this design concept was confirmed by its successful application to diverse bimetallic MOF and PBA precursors, establishing it as a versatile platform for engineering complex carbon architectures. This approach offers a powerful route to materials with precisely controlled structures and surface chemistries, holding significant promise for advanced energy storage, catalysis, and related fields.

## Conflicts of Interest

The authors declare no conflicts of interest.

## Supporting information




**Supporting File**: advs73814‐sup‐0001‐SuppMat.docx.

## Data Availability

The data that support the findings of this study are available from the corresponding author upon reasonable request.
