# Supplementary material for: Vapor‐Assisted Catalysis Enables Precise Construction of MOF‐Derived Hierarchical Carbon Nanoarrays for High‐Performance Supercapacitors
Source: Adv Sci (Weinh). 2026 Jan 11;13(17):e24363. doi: 10.1002/advs.202524363 (PMC13042693; doi:10.1002/advs.202524363)
Supplement: Supplementary file 1 — Supporting File: advs73814‐sup‐0001‐SuppMat.docx. [file ADVS-13-e24363-s001.docx]

**Supporting Information**

**Vapor-Assisted Catalysis Enables Precise Construction of MOF-Derived Hierarchical Carbon Nanoarrays for High-Performance Supercapacitors**

Xiaoyang Deng*, Zihao Wan, Huan Xin, Xiaoguang Wang, Wenjuan Wang*, Feixiong Chen*, Liying Ma, and Naiqin Zhao*

X. Deng, Z. Wan, H. Xin, X. Wang

Laboratory of Advanced Materials and Energy Electrochemistry, Institute of New Carbon Materials, College of Materials Science and Engineering

Taiyuan University of Technology

Taiyuan, Shanxi, 030024, China

E−Mail: dengxiaoyang@tyut.edu.cn

W. Wang

College of Chemistry and Chemical Engineering

Taiyuan University of Technology

Taiyuan, Shanxi, 030024, China

E−Mail: wangwenjuan01@tyut.edu.cn

X. Deng, L. Ma, N. Zhao

School of Materials Science and Engineering,

Tianjin University,

Tianjin 300350, China

E−mail: nqzhao@tju.edu.cn

F. Chen

Disease Networks Research Unit, Faculty of Biochemistry and Molecular Medicine, University of Oulu, 90014 Oulu, Finland

E−mail: feixiong.chen@oulu.fi

1. Experimental Section

**1.1. Preparation of A-ZIF**

**Synthesis of A-ZIF/CC**: Carbon cloth (CC, 1 cm*1.5 cm) was first cleaned by sonication in acetone, ethanol, and deionized water, followed by treatment in concentrated nitric acid. The treated CC was immersed in an aqueous solution of Co(NO_3_)_2_·6H_2_O (40 mL, 0.592 g). Separately, a 2-methylimidazole (2-mIm) aqueous solution (40 mL, 1.312 g) was prepared. The 2-mIm solution was added to the Co(NO_3_)_2_ solution, and the pre-soaked CC was immediately immersed in the resulting mixture. The reaction proceeded for 4 h at room temperature. The product, A-ZIF/CC, was then collected, washed thoroughly with deionized water, and dried in an oven at 60 ^o^C overnight.

**Synthesis of Bimetallic Arrays (A-CoM-ZIF/CC, M = Cu, Ni, Zn)**: The procedure was identical to the synthesis of A-ZIF/CC, except that the Co(NO_3_)_2_ solution was replaced with a mixed-metal salt solution. Specifically:

CoCu-ZIF: 0.437 g of Co(NO_3_)_2_·6H_2_O and 0.121 g of Cu(NO_3_)_2_·6H_2_O were used.

CoNi-ZIF: 0.437 g of Co(NO_3_)_2_·6H_2_O and 0.145 g of Ni(NO_3_)_2_·6H_2_O were used.

CoZn-ZIF: 0.437 g of Co(NO_3_)_2_·6H_2_O and 0.148 g of Zn(NO_3_)_2_·6H_2_O were used.

**1.2. Synthesis of MOF Powder Auxiliaries**

**Synthesis of P-ZIF(Zn):** A methanolic solution of 2-mIm (200 mL, 6.49 g) was rapidly poured into a methanolic solution of Zn(NO_3_)_2_·6H_2_O (200 mL, 2.97 g) under vigorous magnetic stirring at room temperature. The reaction was maintained for 1 h. The resulting white powder was collected by centrifugation, washed three times with methanol, and dried under vacuum at 60 °C.

**Synthesis of P-ZIF(Co) and P-ZIF(ZnCo):** These powders were synthesized using an analogous procedure, with the metal salt solution modified as follows:

For P-ZIF(Co): 2.92 g of Co(NO_3_)_2_·6H_2_O was used.

For P-ZIF(ZnCo): 1.48 g of Zn(NO_3_)_2_·6H_2_O and 1.46 g of Co(NO_3_)_2_·6H_2_O were used.

**1.3. Synthesis of Hierarchical Carbon Arrays (HCAs)**

A piece of A-ZIF/CC was placed horizontally in a ceramic boat above, but not in physical contact with, 200 mg of P-ZIF(Zn) powder. The boat was placed in a tube furnace and heated to 800 °C at a ramp rate of 2 °C min^-1^ under an Ar flow, and held for 2 h. After cooling to room temperature, the as-pyrolyzed material was etched with 3M HCl at 60 °C for 12 h to remove accessible metal particles. The final product, HCA800, was obtained after washing with deionized water until neutral and drying.

**Temperature Variation:** HCA700 and HCA900 were synthesized under identical conditions, except the pyrolysis temperatures were 700 °C and 900 °C, respectively.

**Control Samples:** SCA800 was synthesized by pyrolyzing A-ZIF/CC at 800 °C using P-ZIF(Co) as the auxiliary. Other control experiments were conducted by replacing the P-ZIF(Zn) auxiliary with nano-Zn powder, nano-ZnO powder, or melamine. When using the A-CoCu-ZIF, A-CoNi-ZIF and A-CoZn-ZIF instead of A-ZIF, the CoCu-HCA, CoNi-HCA and CoZn-HCA can be obtained.

**1.4. Synthesis of PBA-Derived CNT Arrays**

**Synthesis of Co-Co-PBA and Co-Ni-PBA Precursor:** Typically, 100 mL of an aqueous solution of K_3_[Co(CN)_6_] (1.0 mmol) was added to 100 mL of an aqueous solution containing Co(NO_3_)_2_·6H_2_O (3.0 mmol) or Ni(NO_3_)_2_·6H_2_O (3.0 mmol) under stirring, respectively. After stirred at room temperature for 60 s, two pieces of carbon cloth were placed into above mixture solution and aged for 24 h at room temperature. Finally, the obtained CC-supported products were washed with deionized water followed by a drying treatment in an oven at 65 ^o^C for 12 h, and they were denoted as Co-Co-PBA and Co-Ni-PBA, respectively.

**Pyrolysis:** The Co-Co-PBA was pyrolyzed at 800 °C under an Ar atmosphere to obtain the Co-CNT under identical conditions as HCA800. The synthesis of CoNi-CNT from Co-Ni-PBA followed a similar procedure.

**1.5. Fabrication of Flexible Symmetric Supercapacitor (FSC)**

Preparation of PVA/KOH Gel Electrolyte: Polyvinyl alcohol (PVA) gel electrolyte was prepared by dissolving 5 g PVA was dissolved in 45 mL of deionized water, followed by a vigorous stirring at 95 °C for at least one hour, after which the bubbles were removed under vacuum at room temperature. The resulting solution was poured into a culture dish and subjected to freeze at −3 °C for 6 h. Before assembling the FSC, the PVA gel was immersed in 6M KOH electrolyte to obtain the PVA/KOH gel electrolyte.

**Fabrication of FSC**: Typically, two pieces of HCA800 (1 cm*2 cm, with an active material mass of ~1.2 mg each) was were soaked in the 6M KOH electrolyte for 5 min. The symmetric flexible supercapacitor was then assembled by overlapping the two HCA800 electrodes, with a thin layer of PVA/KOH gel electrolyte sandwiched between them.

**2. Materials Characterization**

The morphologies and structure were investigated by scanning electron microscopy (SEM; Hitachi S4800/S7800) and transmission electron microscopy (TEM; FEI Tecnai G2 F20 TEM). Crystal structures were analyzed by X-ray diffraction (XRD; Rigaku D/max-2500 with Cu-Kα radiation). Raman spectra were recorded on a LabRAM HR Raman spectrometer with a 532 nm laser. N_2_ adsorption-desorption isotherms were measured at 77 K using a Micromeritics ASAP 2020 analyzer. X-ray photoelectron spectroscopy (XPS) was performed on a PHI Quantera SXM system. Thermogravimetric analysis coupled with mass spectrometry (TG-DSC-MS) was conducted on an STA449F3 instrument under an Ar atmosphere. In-situ Raman spectroscopy during electrochemical cycling was performed on a Renishaw Qontor with an excitation wavelength of 532 nm.

**3. Supercapacitor measurement**

**Three-electrode system:** Measurements were performed in a 6 M KOH aqueous electrolyte using the HCA/CC samples as the working electrode, a platinum foil as the counter electrode, and a Hg/HgO electrode as the reference electrode. The mass loading of the active material was ~1.2 mg cm^-2^.

**Two-electrode System:** Two-electrode symmetrical supercapacitors was assembled with two pieces of electrode in aqueous electrolyte. And the device was assembled as described in Section 1.5.

**Calculations:** The specific capacitances were calculated from the galvanometric charge-discharge curves using the following equations: C= *I∆t*/*m∆V* (three-electrode system) and C= *I∆t*/*m∆V* (two-electrode system), where C (F g^-1^) is the specific capacitance, *I* (A) is the constant current, Δ*t* (s) is the discharge time at corresponding current density, *m* (g) is the mass of active material, and the Δ*V* (V) is the potential (voltage) window. Energy density and power density for the supercapacitor were calculated using the following equations: E=1/2*C*(*∆V*)^2^ and P=3600E/*∆t*, where *E* (Wh kg^-1^) is the energy density, *P* (W kg^-1^) is the power density, C (F g^-1^) is the specific capacitance of the electrode, Δ*V* (V) is the potential window, and Δ*t* (s) is the discharge time.

**4. Computational and Calculation Details**

**4.1. DFT Computational Details**

Density functional theory (DFT) calculations were performed using the Vienna Ab-initio Simulation Package (VASP). The projector augmented wave (PAW) method and the Perdew–Burke–Ernzerhof (PBE) generalized gradient approximation (GGA) exchange-correlation functional were employed. The van der Waals interactions were included by using DFT-D3 method. To sample the Brillouin zone, the k point was set to 4 × 4 × 1 gamma point grid, with a cut-off energy of the plane wave set to 450 eV. The convergence criterion of the energy calculation and force calculation were 10^−5^ eV and 0.02 eV Å^−1^, respectively. A vacuum layer of 15 Å was applied to prevent periodic interactions. Spin-polarized calculations were conducted for systems containing transition metal atoms. The adsorption energy (*E_ads_*) was calculated as follow:

*E_ads_*=*E_total_*−*E_substrate_*−*E_adsorbate_*

**4.2. Specific surface area calculation**

The intrinsic specific surface area of the HCA material (*S_HCA_*) was deconvoluted from the total measured surface area of the composite (*S_total_*) using the following equation, accounting for the contribution of the bare carbon cloth substrate (*S_CC_*):

*S_HCA_* = [ (*S_total_*×*m_total_*)-( *S_CC_*× *m_CC_*) ] / *m_HCA_*

Where*m_total_*, *m_CC_*, and *m_HCA_* are the masses of the composite, the bare CC, and the HCA active material, respectively. Using the measured values (*m_CC_* = 12 mg, *m_HCA_* = 1.2 mg, *S_CC_* ≈ 3.5 m^2^ g^-1^, *S_total_* = 53.1 m^2^ g^-1^), the specific surface area of HCA800 was calculated to be 549.1 m^2^ g^-1^.

The entire electrode (including the carbon cloth substrate) exhibited a BET specific surface area of 53.10 m^2^ g^-1^. Given the low surface area of the carbon cloth (~1-3 m^2^ g^-1^) and its significant mass fraction, the intrinsic specific surface area of the active HCA800 material was estimated to be approximately 549 m^2^ g^-1^ after normalization.

**4.3. Ion Diffusion Coefficient**

The diffusion coefficient (D) can be calculated from the following equation:

$$D=\frac{R^{2}T^{2}}{2A^{2}n^{4}F^{4}C^{2}\sigma^{2}}$$

Where *R* is the gas constant, *T* is the absolute testing temperature, *A* is the surface area of the electrode, *n* is the number of electrons transferred, *F* is the Faraday constant, *C* is the ions concentration, and σ is the Warburg factor. The σ can be obtained by a linear fit between Z’ and σ ^−1/2^.

**4.4. Quantum Capacitance**

Based on the DFT-calculated density of states (DOS), the quantum capacitance *C_q_* of the different electrode models was further evaluated, and the quantum capacitance can be expressed as:

C_q_ = e^2^·DOS(EF)

where DOS(E_F_) is the density of states at the Fermi level and and *e* is the elementary charge. Considering the quasi-two-dimensional nature of the systems, *Cq*​ was normalized by the in-plane area of the simulation cell.

According to the DFT calculation results, the DOS at the Fermi level for the pristine carbon matrix (C) is relatively low, corresponding to a quantum capacitance of only 0.00117 µF cm^-2^. Upon anchoring Co nanoparticles on carbon (Co/C), D (E_F_) increases markedly, and quantum capacitance rises to 0.395 µF cm^-2^. Further N,O co-doping of the carbon support combined with Co nanoparticle (Co/N,O–C) slightly enhances D (E_F_) compared with Co/C, yielding the highest quantum capacitance of 0.407 µF cm^-2^. In contrast, the N,O co-doped carbon without Co (N,O–C) exhibits an intermediate quantum capacitance of 0.152 µF cm^-2^, lying between that of pristine C and the Co-containing systems.

These results indicate that N,O heteroatom doping effectively modulates the carrier density and increases the DOS near the Fermi level, thereby improving the quantum capacitance. More importantly, the introduction of Co species contributes even more significantly to the DOS at E_F_​, and the synergistic interaction between Co and N,O dopants leads to the highest DOS and quantum capacitance for the Co/N,O–C electrode. This theoretical trend is consistent with experimental observations, where the Co/N,O–C electrode exhibits enhanced specific capacitance and superior rate performance compared with the undoped or Co-free counterparts.

Total capacitance analysis: The total capacitance (C_total_) follows: 1/C_total_ = 1/C_q_ + 1/C_EDL_, where C_EDL_ is the electrical double-layer capacitance. Due to the high C_EDL_ from HCA electrodes, the limitations influence from quantum capacitance was greatly reduced.

5. Supporting Figures


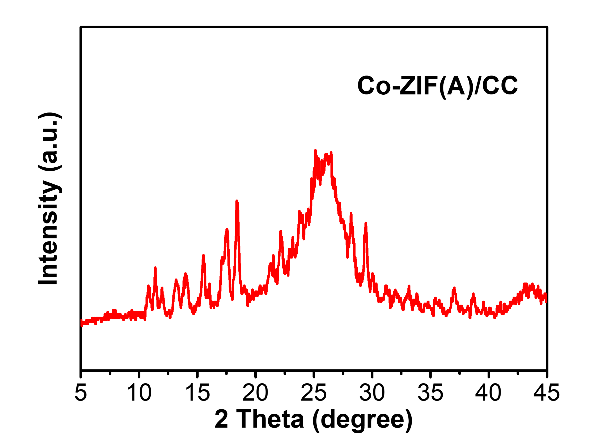


**Figure S1. XRD patterns of the as-synthesized A-ZIF precursor grown on carbon cloth (A-ZIF/CC).**


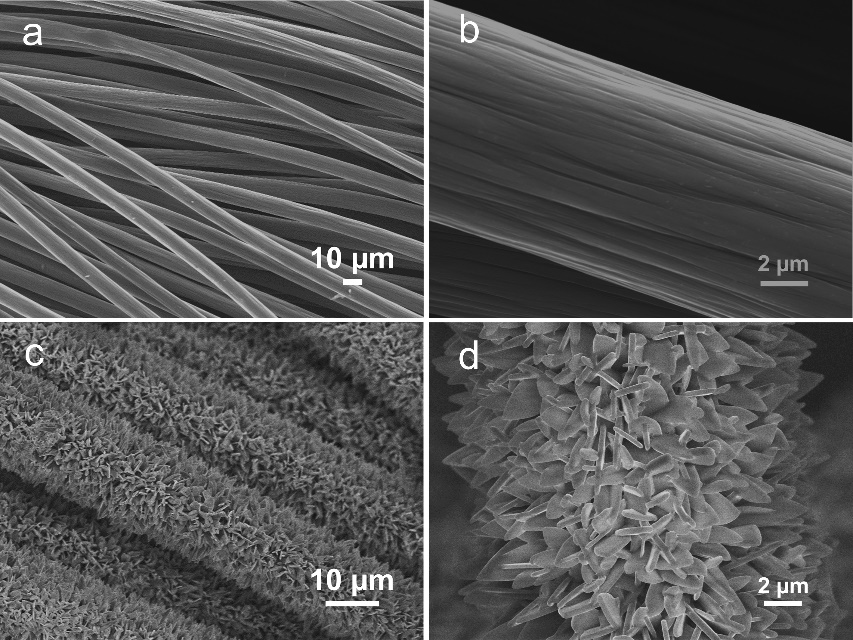


**Figure S2. SEM images of (a) the bare carbon cloth (CC) substrate and (b) the A-ZIF nanosheet array grown on the CC substrate (A-ZIF/CC).** The images show the smooth surface of the individual carbon fibers in the bare cloth and the uniform, dense coverage of the nanosheet array after MOF growth.


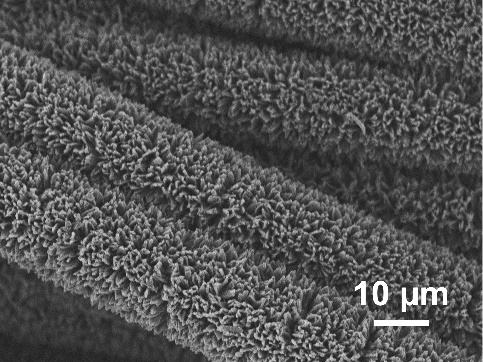


**Figure S3. Low-magnification SEM image of HCA800.** This overview image demonstrates the large-area uniformity of the hierarchical array architecture across the entire carbon cloth substrate after pyrolysis and etching.


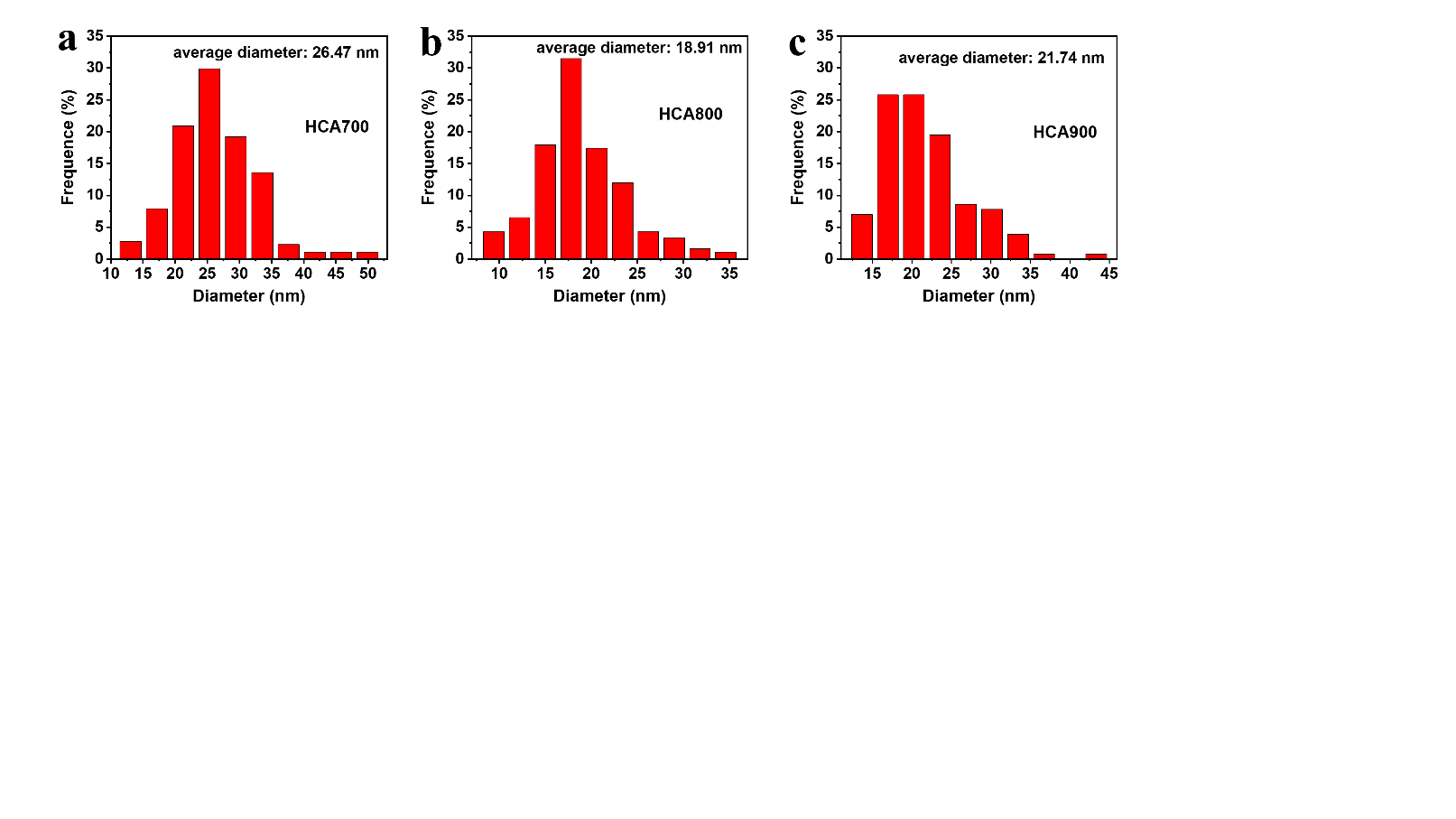


**Figure S4. CNT diameter distribution histograms for (a) HCA700, (b) HCA800, and (c) HCA900.** The statistics were derived from their corresponding SEM images. The average CNT diameter is ~26 nm for HCA700, ~19 nm for HCA800, and ~21 nm for HCA900, indicating that the pyrolysis temperature influences the catalyst particle size and subsequent CNT diameter.


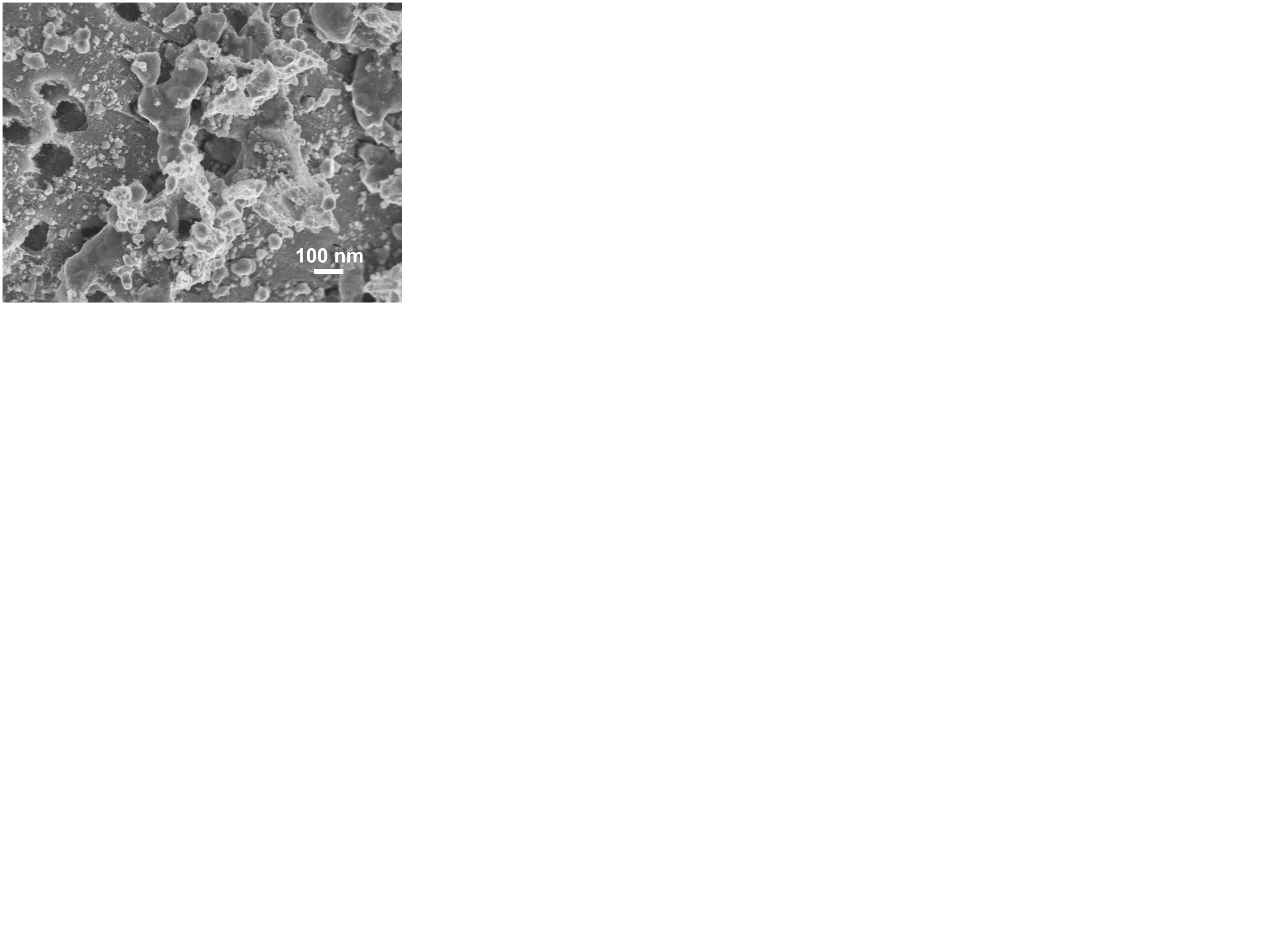


**Figure S5. SEM images of the material obtained from direct pyrolysis of A-ZIF/CC at 800 °C without any auxiliary.** The images reveal the complete collapse of the nanosheet architecture and the formation of large, sintered metal particles (bright spots). This result highlights the essential role of the vapor-phase regulation in preserving the precursor's morphology.


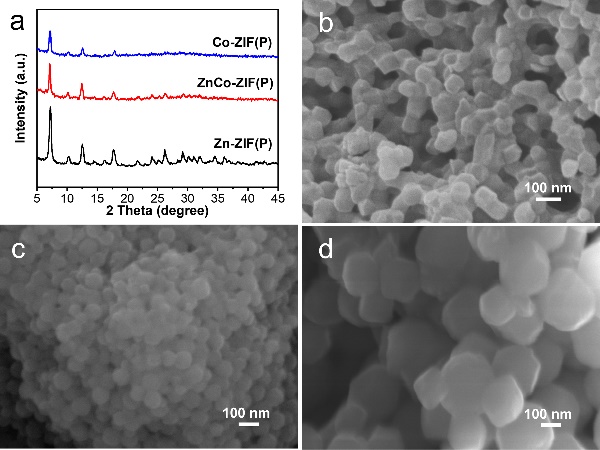


**Figure S6. (a) XRD patterns and (b-d) SEM images of the synthesized auxiliary powders: (b) P-ZIF(Zn), (c) P-ZIF(ZnCo), and (d) P-ZIF(Co).** The XRD patterns confirm the successful synthesis of the intended crystalline ZIF structures. The SEM images show that all auxiliary powders consist of uniform, polyhedral nanoparticles.


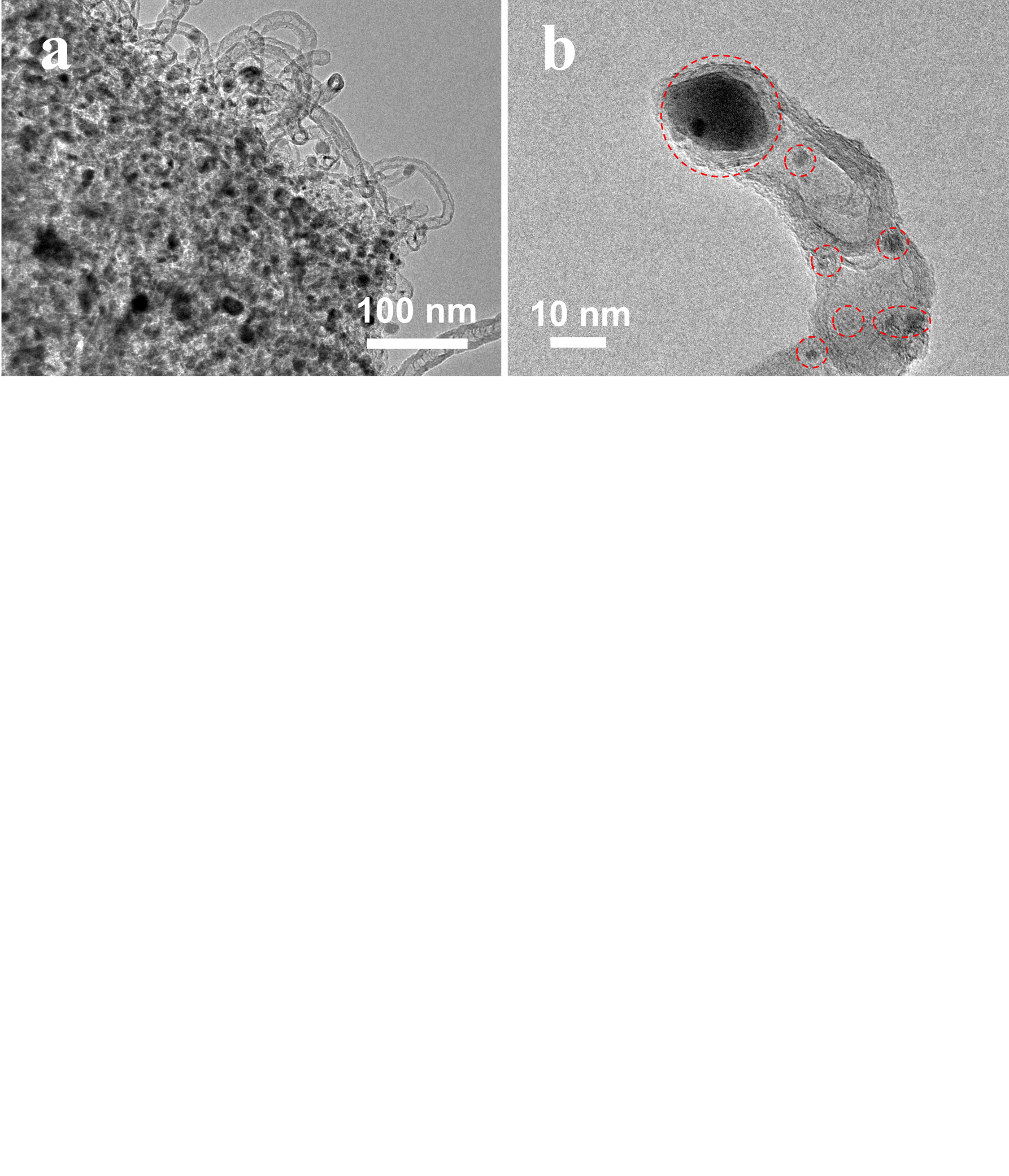


**Figure S7. TEM images of HCA800 before acid-etching.** The HCA800 sample before etching showed very small cobalt particles with no significant aggregation. Simultaneously, HRTEM imaging revealed minute nanoparticles on the carbon nanotube walls, which are suggested to be either unagglomerated cobalt or evaporated zinc particles.


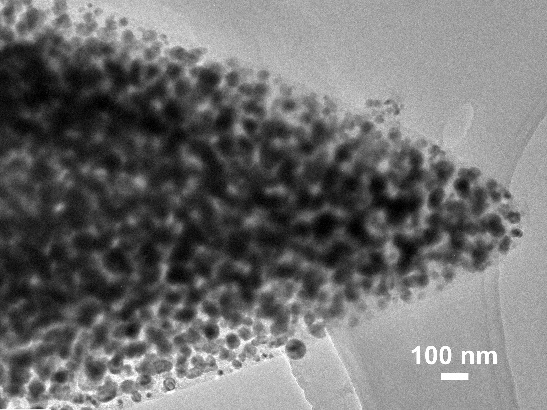


**Figure S8. TEM images of SCA800, the material obtained using P-ZIF(Co) as the auxiliary.** The images show severe structural collapse, nanosheet degradation, and large cobalt agglomerates, confirming that a cobalt-based vapor source alone is detrimental to the architecture.


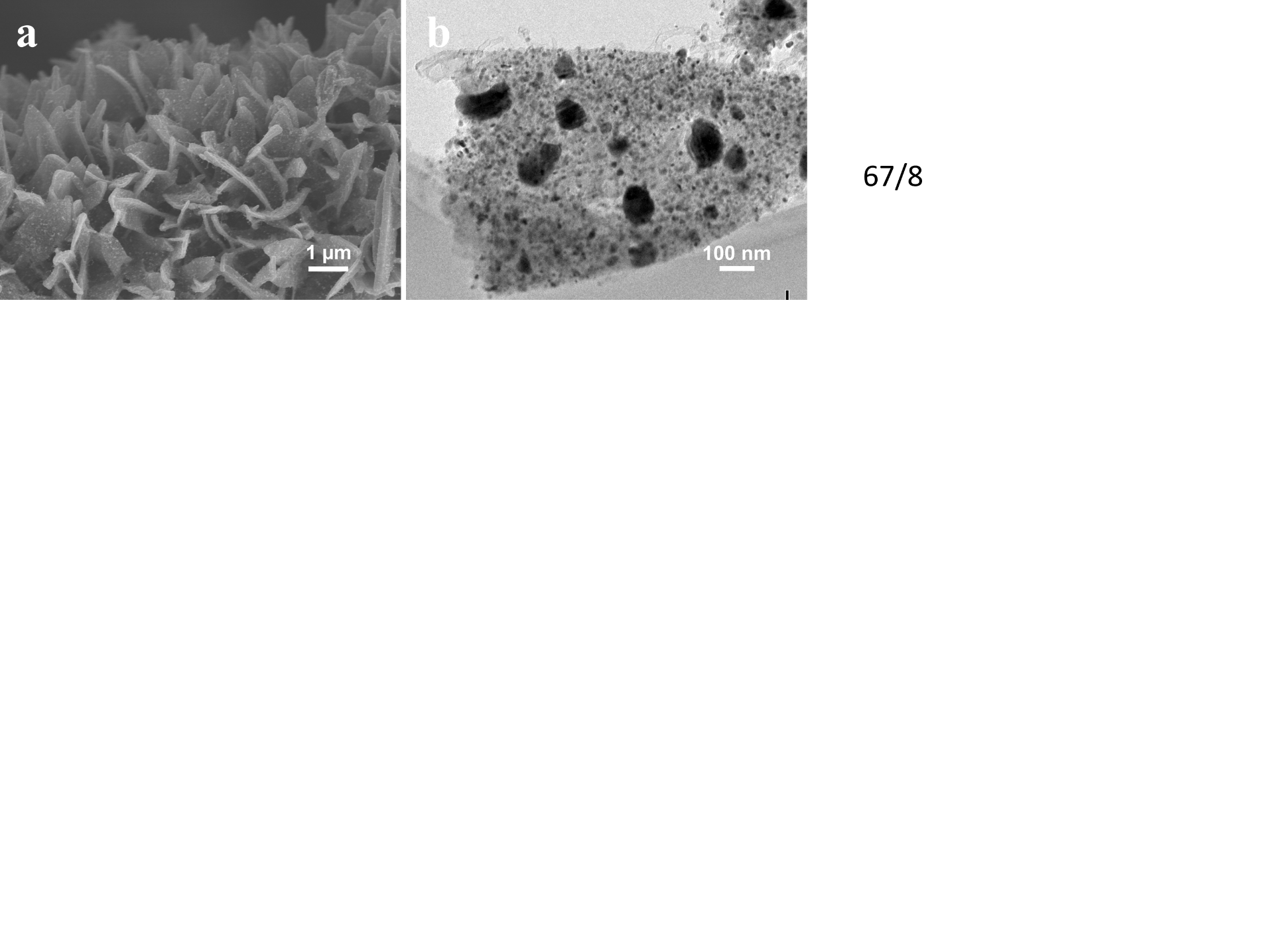


**Figure S9. SEM and TEM images of the material obtained using the bimetallic P-ZIF(ZnCo) auxiliary.** The morphology is well-preserved, and nascent CNTs are observed. This result, when compared with Figure S7, strongly suggests that zinc is the key element responsible for structural preservation and initiating CNT growth.


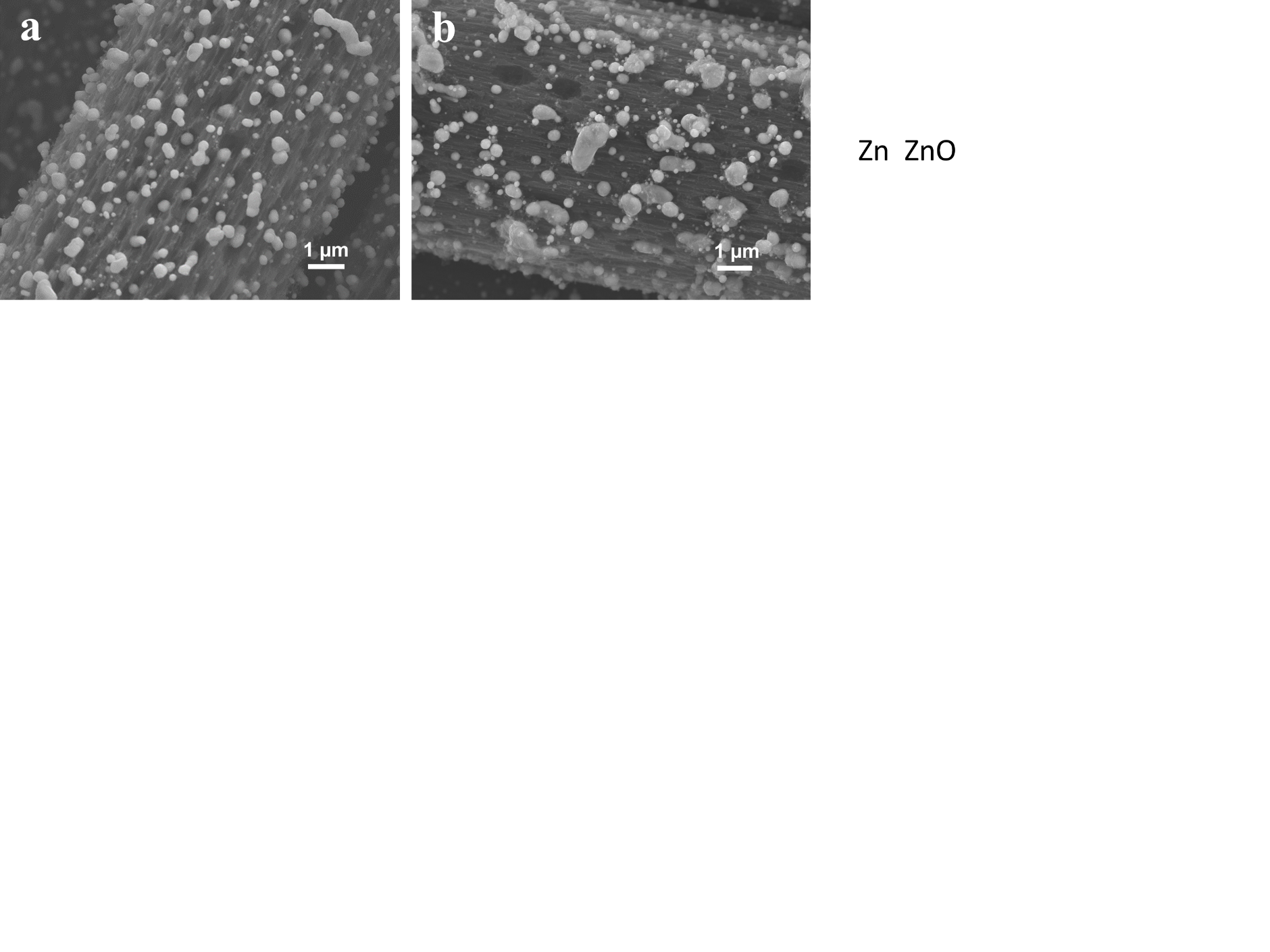


**Figure S10. SEM images of control experiments using alternative auxiliaries. (a) nano-Zn powder and (b) nano-ZnO powder.**


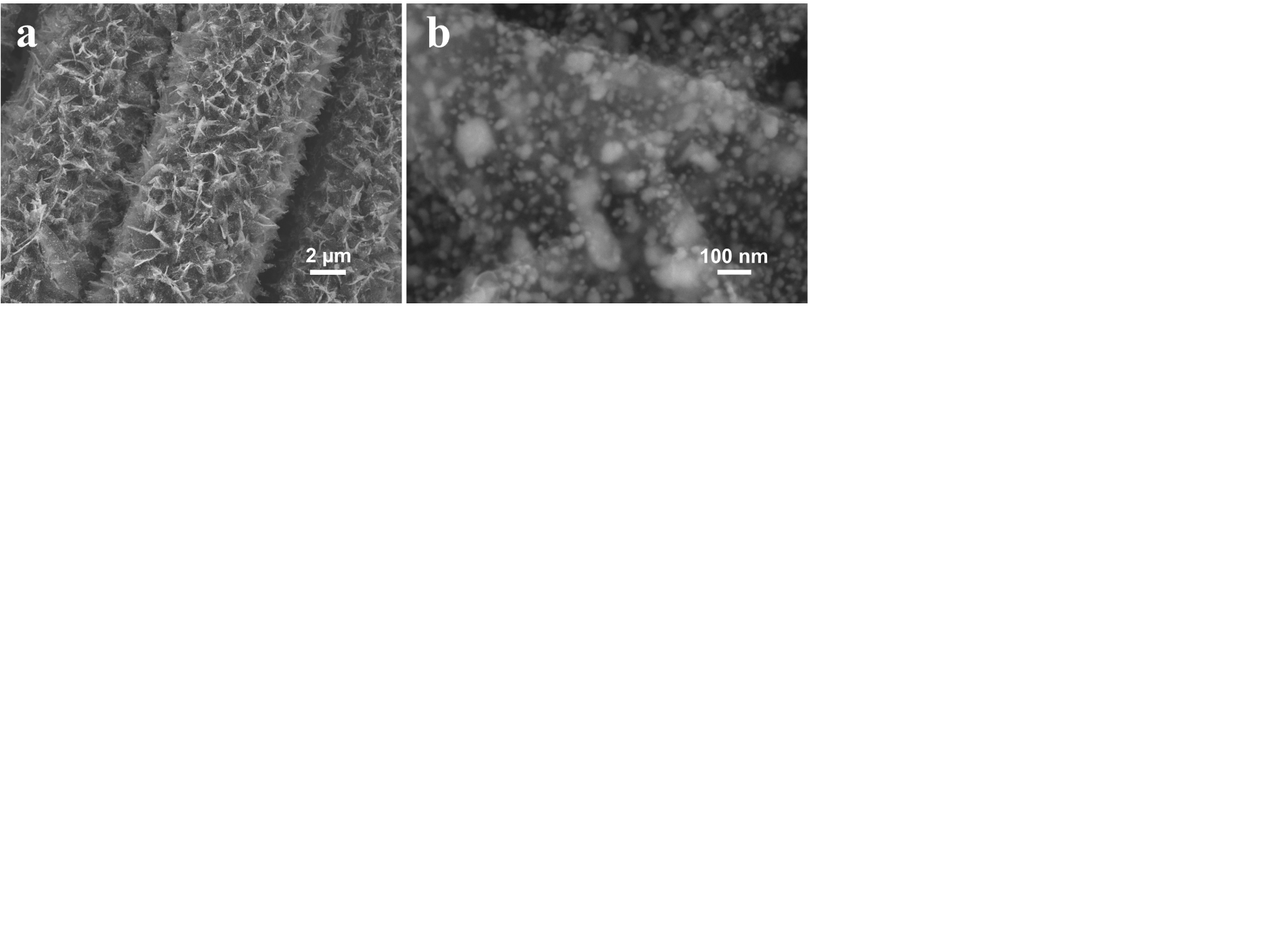


**Figure S11. SEM images of control experiments using a mixture of nano-Zn powder and P-ZIF(Co).**

When common zinc sources like nano-Zn or nano-ZnO powders were substituted, the synthesis failed catastrophically, leading to structural collapse and aggressive etching (Figure S10). Even more tellingly, physically mixing nano-Zn powder with P-ZIF(Co), an auxiliary agent known to produce gases, yielded the exact same outcome as P-ZIF(Co) alone: ultrathin nanosheets with a complete absence of CNTs (Figure S11). These failures build an irrefutable case that the unique, periodic structure of Zn-ZIF is the master orchestrator of the timely and effective generation and release of fine zinc vapor. We posit that the periodic structure formed by zinc ions and organic ligands within the ZIF framework promotes the generation of fine zinc vapor, capable of volatilizing at temperatures below the theoretical boiling point of zinc. This fine, controlled zinc vapor flux is essential for its participation in the comprehensive processes of nanosheet microstructure formation, array morphology maintenance, and carbon nanotube array generation.


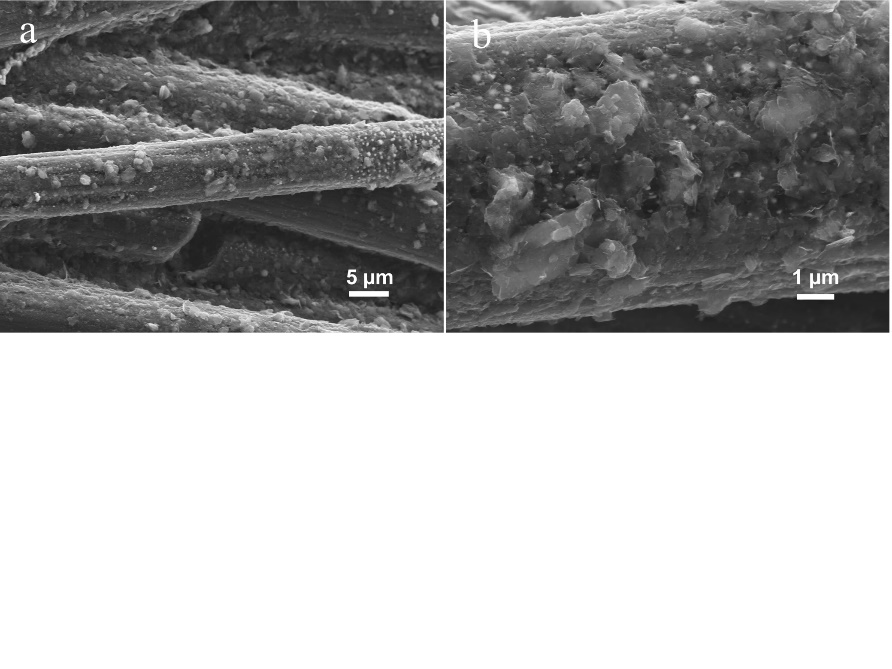


**Figure S12. SEM images of control experiments using the melamine.** Melamine, a common auxiliary agent for CNT growth in the field of MOFs, failed to preserve the vital nanosheet array, though it did mitigate fiber damage.


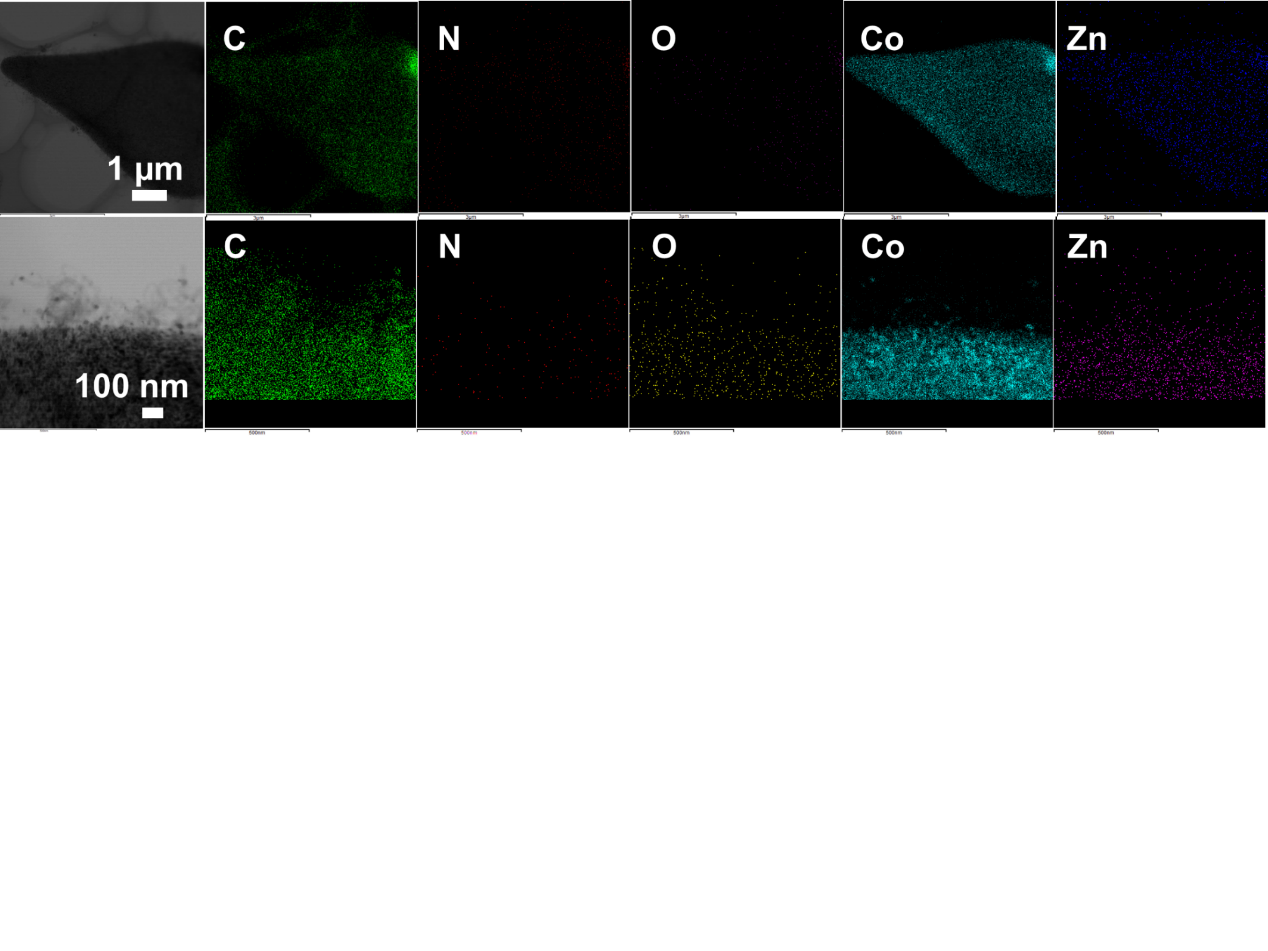


**Figure S13. TEM image and corresponding elemental mapping (C, N, O, Co, Zn) of HCA800 before acid etching.** The uniform distribution of all elements, particularly Zn, across the nanosheet array indicates that the zinc vapor effectively permeates and interacts with the entire precursor structure during pyrolysis.


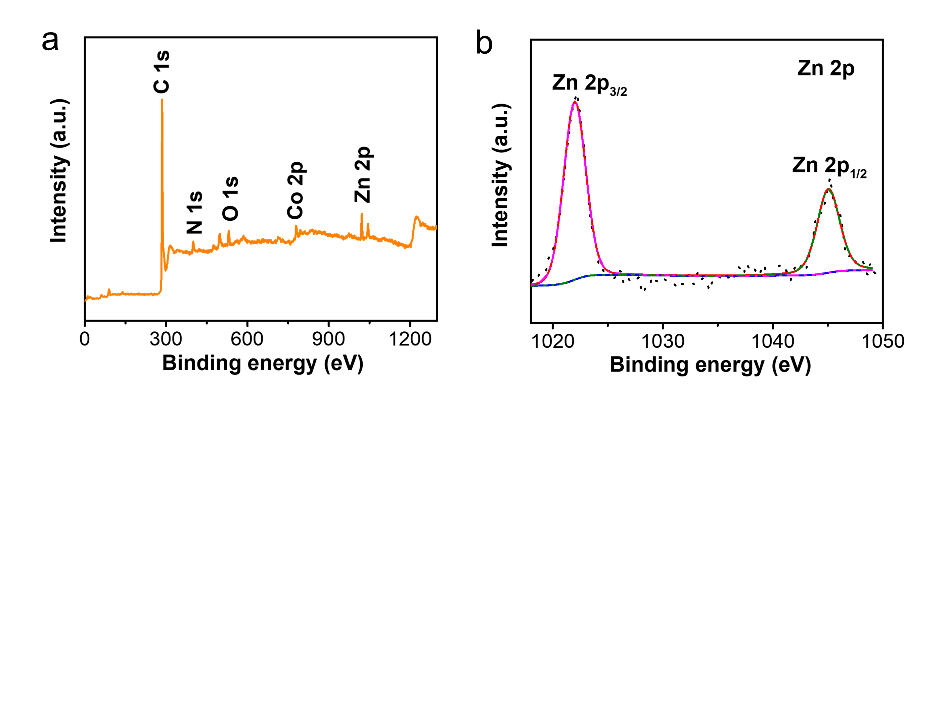


**Figure S14. XPS spectra of HCA800 before acid etching.** (a) Survey spectrum and (b) high-resolution Zn 2p spectrum. The Zn 2p spectrum, with peaks at binding energies characteristic of metallic zinc (Zn^0^), confirms that zinc acts as a transient, physical mediator and is not chemically incorporated as an oxide or other compound.


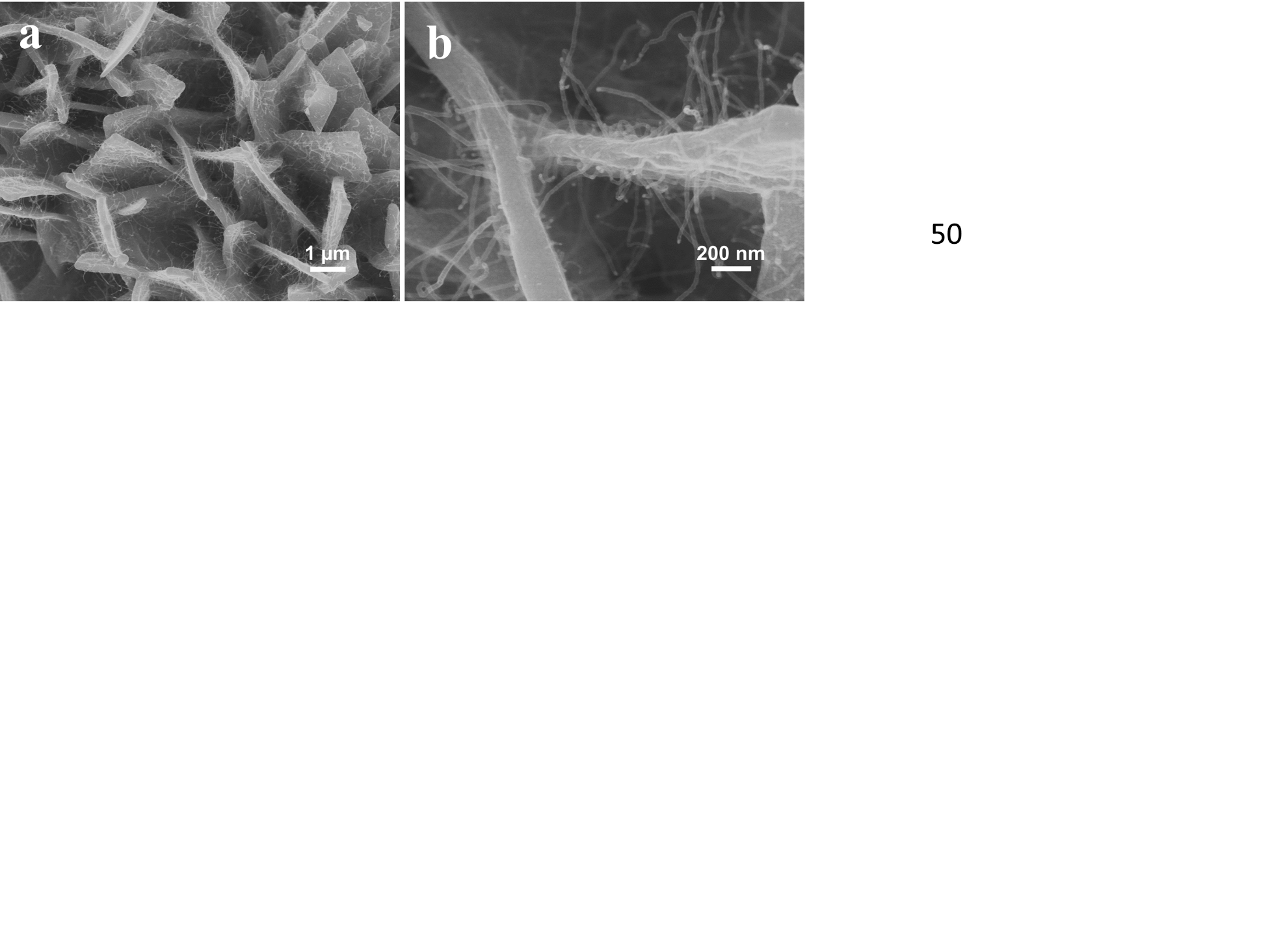


**Figure S15. SEM images of the material synthesized using a reduced amount (100 mg) of P-ZIF(Zn) auxiliary.** While the nanosheet structure is partially preserved, CNT growth is sparse. This indicates that a sufficient flux of Zn vapor is necessary for effective catalytic CNT growth.


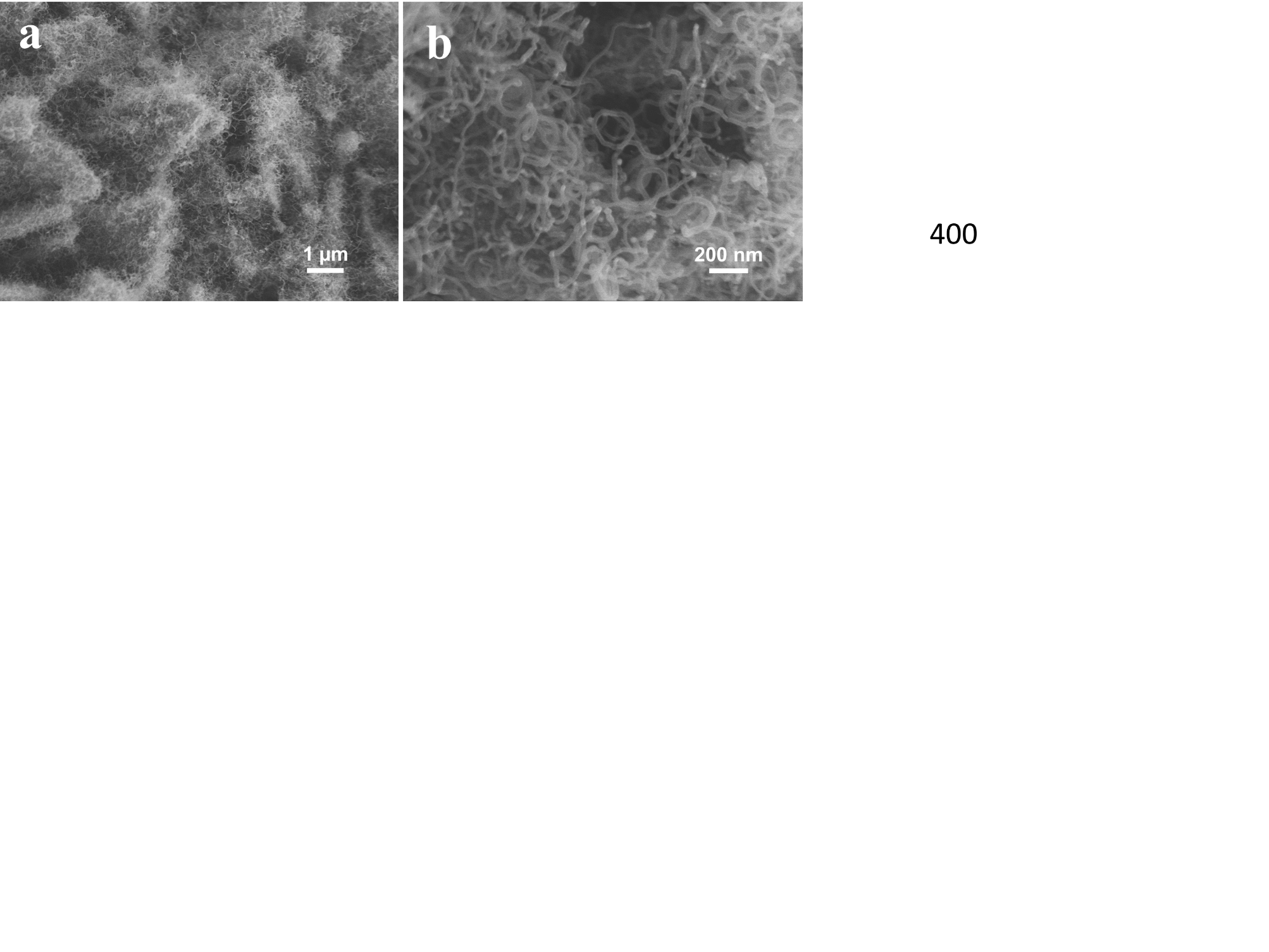


**Figure S16. SEM images of the material synthesized using an excess amount (400 mg) of P-ZIF(Zn) auxiliary.** The material shows a dense coverage of CNTs, but they are significantly shorter and thicker compared to HCA800. This suggests that an excessive Zn vapor flux may lead to partial passivation of the Co catalyst, inhibiting sustained CNT growth, consistent with DFT results.


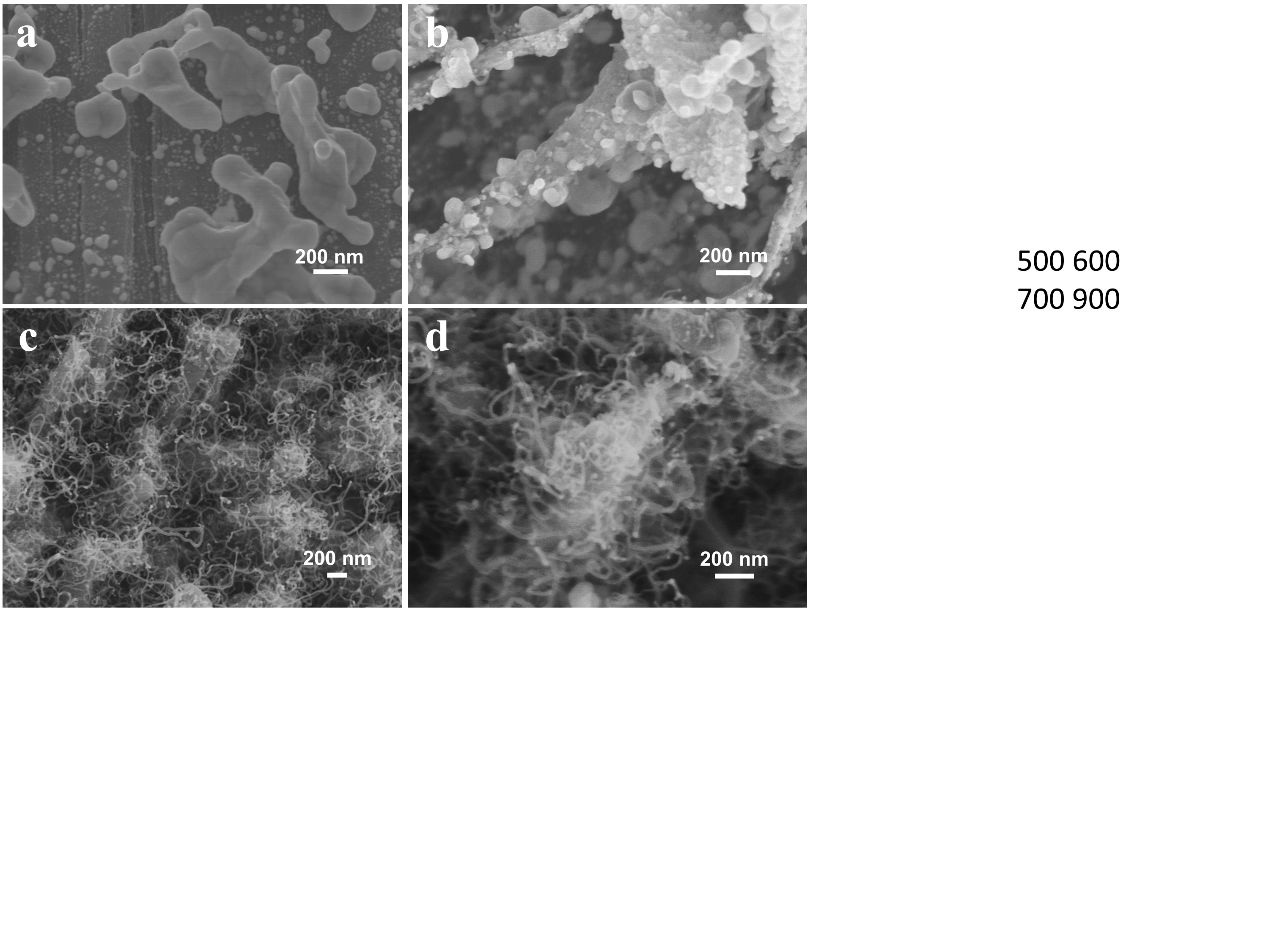


**Figure S17. Morphological evolution of the HCA material as a function of pyrolysis temperature.** SEM images of samples prepared at (a) 500 °C, (b) 600 °C, (c) 700 °C, and (d) 900 °C. To map the morphological evolution as a function of temperature under vapor-assisted pyrolysis, a systematic study was conducted from 500 to 900 °C, yielding samples designated HCA500, HCA600, HCA700, and HCA900, respectively. The results reveal a clear, temperature-driven pathway to the desired hierarchical architecture. At a modest 500 °C, the delicate nanosheet architecture disintegrated into irregular particles, a result of premature structural collapse as organic linkers and guest molecules were removed (Figure 2c and S16a). A significant improvement was observed at 600 °C, where the array structure was robustly maintained, though decorated with large and agglomerated cobalt particles (Figure 2d and S16b). The optimal conditions for hierarchical structure formation emerged at 700 °C. HCA700 showcased the desired and well-preserved nanosheet array, which now served as a scaffold for a dense and interwoven CNT network (Figure 2e). These nanotubes, originating from the nanosheets themselves, exhibited an average diameter of ~26 nm (Figure S4a). At 800 °C (HCA800), further optimization of CNT density and morphology was achieved, leading to the best electrochemical performance. Pushing the temperature further to 900 °C, HCA900 retained the array and even featured a higher density of CNTs with a smaller diameter (about 21 nm), but the length of CNTs was drastically curtailed (Figure 2f and S16d).


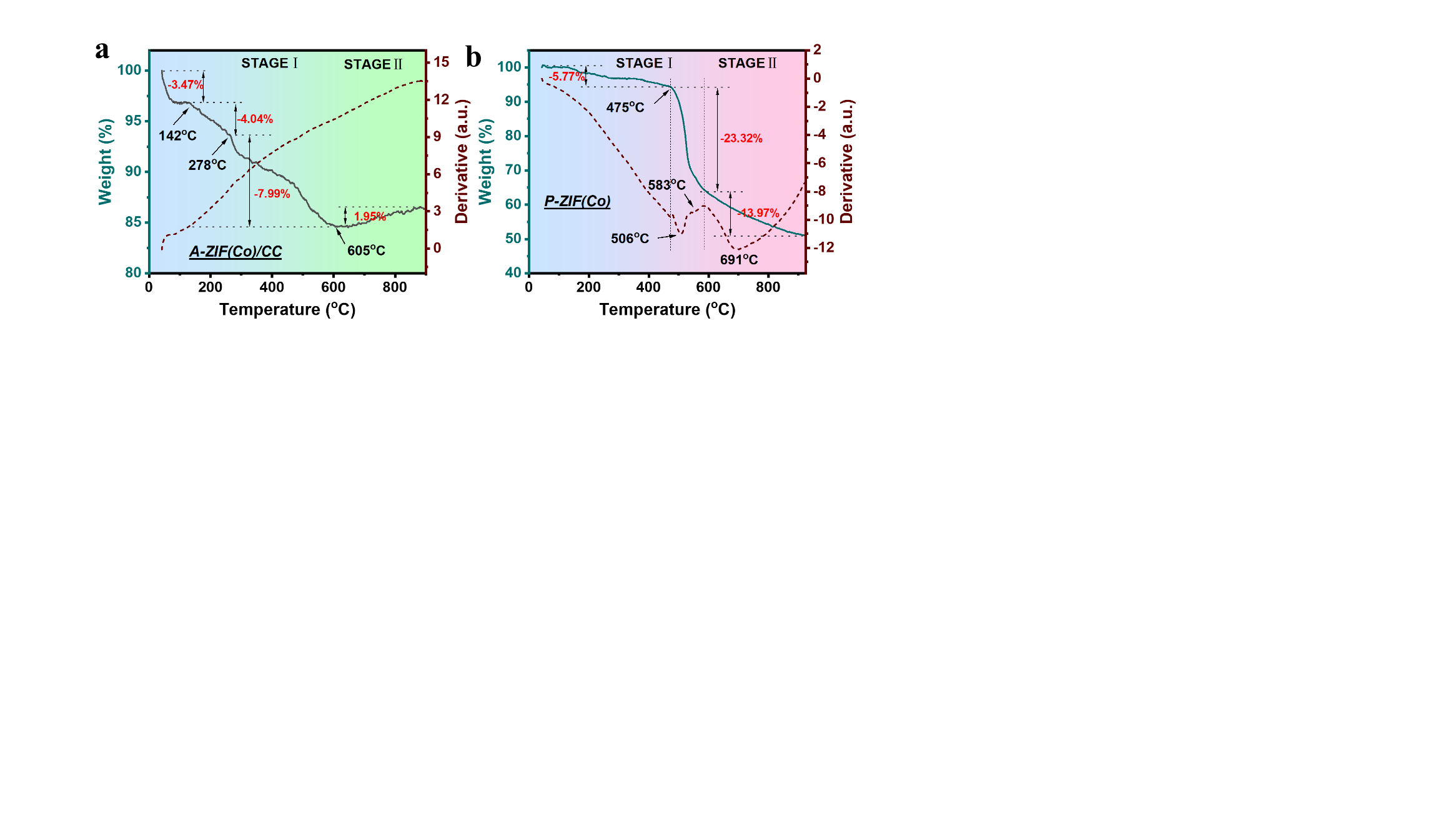


**Figure S18. TG/DSC curves of (a) the A-ZIF precursor and (b) the P-ZIF(Co) auxiliary.** The similar decomposition temperature ranges highlight the thermal synchronicity between the precursor and auxiliaries. This timing is crucial, ensuring the protective vapor is released precisely when the A-ZIF framework becomes thermally vulnerable.


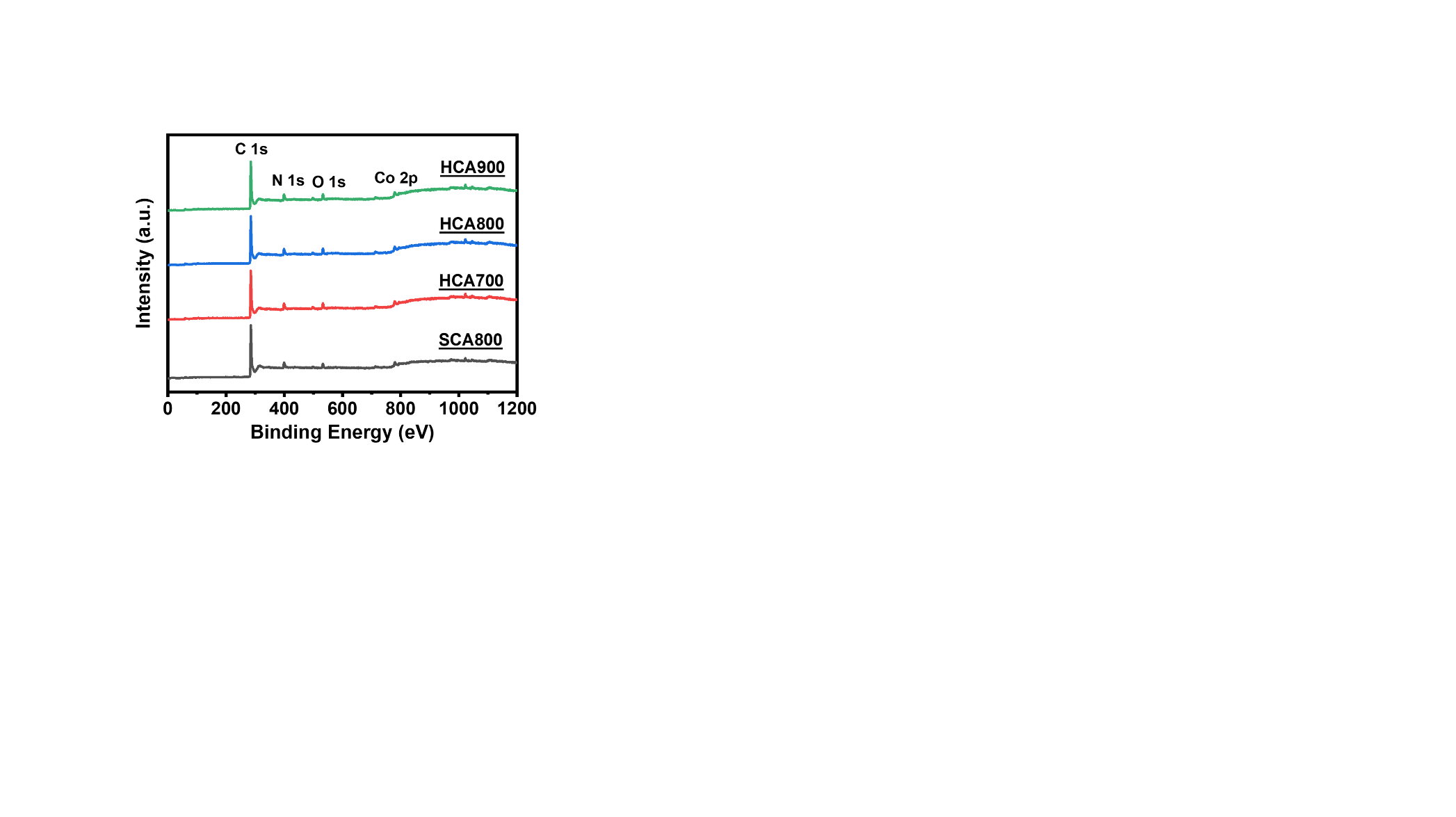


**Figure S19. Full-range XPS survey spectra of the HCA series (HCA700, HCA800, HCA900) and the control sample SCA800.** The spectra confirm the presence of C, N, O, and Co in all samples after pyrolysis and acid etching.


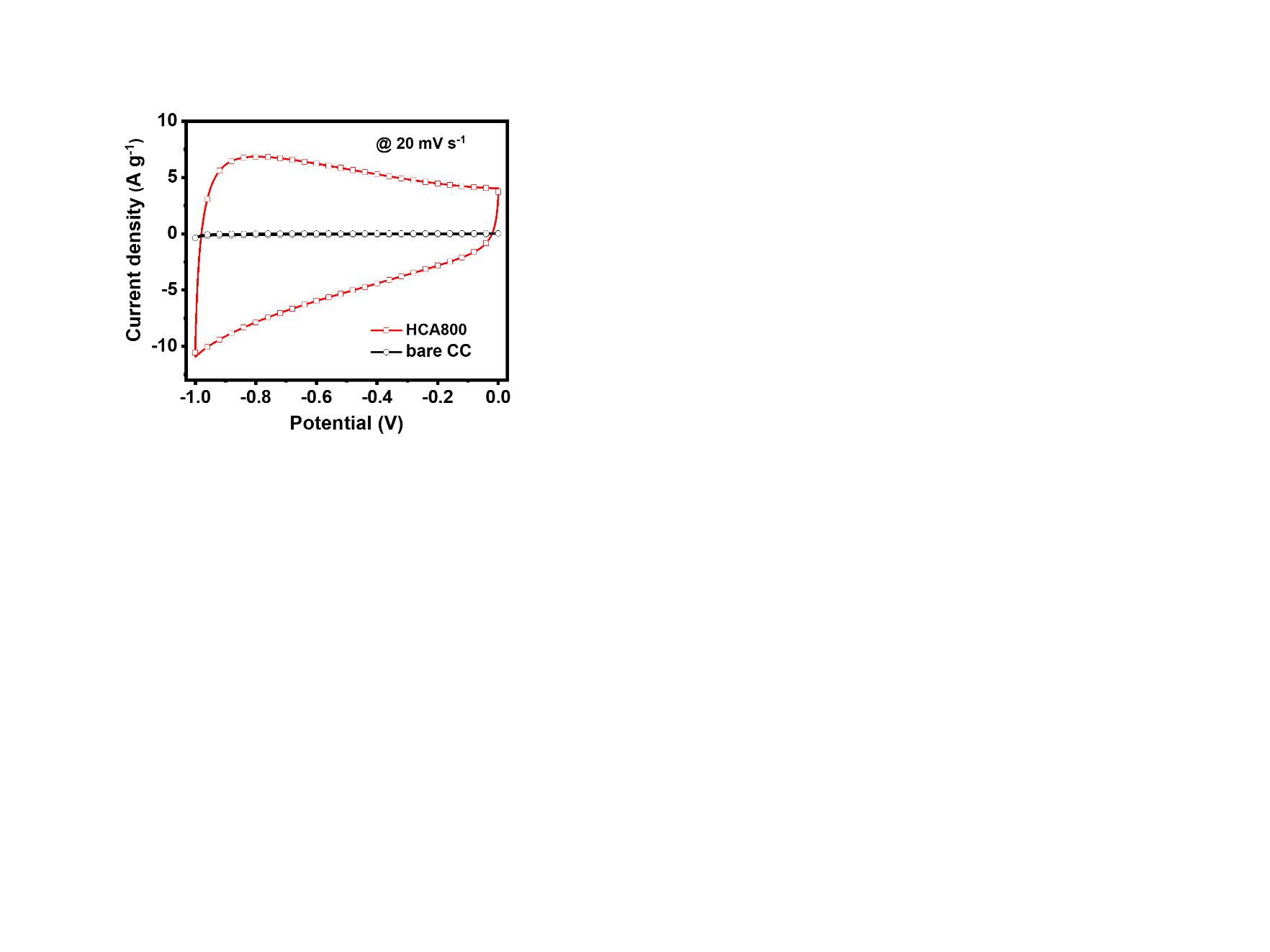


**Figure S20. CV curves of the HCA800 electrode and a bare carbon cloth (CC) electrode at a scan rate of 20 mV s^-1^.** The negligible current response from the bare CC confirms that its capacitive contribution to the overall performance of the HCA800/CC composite electrode is insignificant.


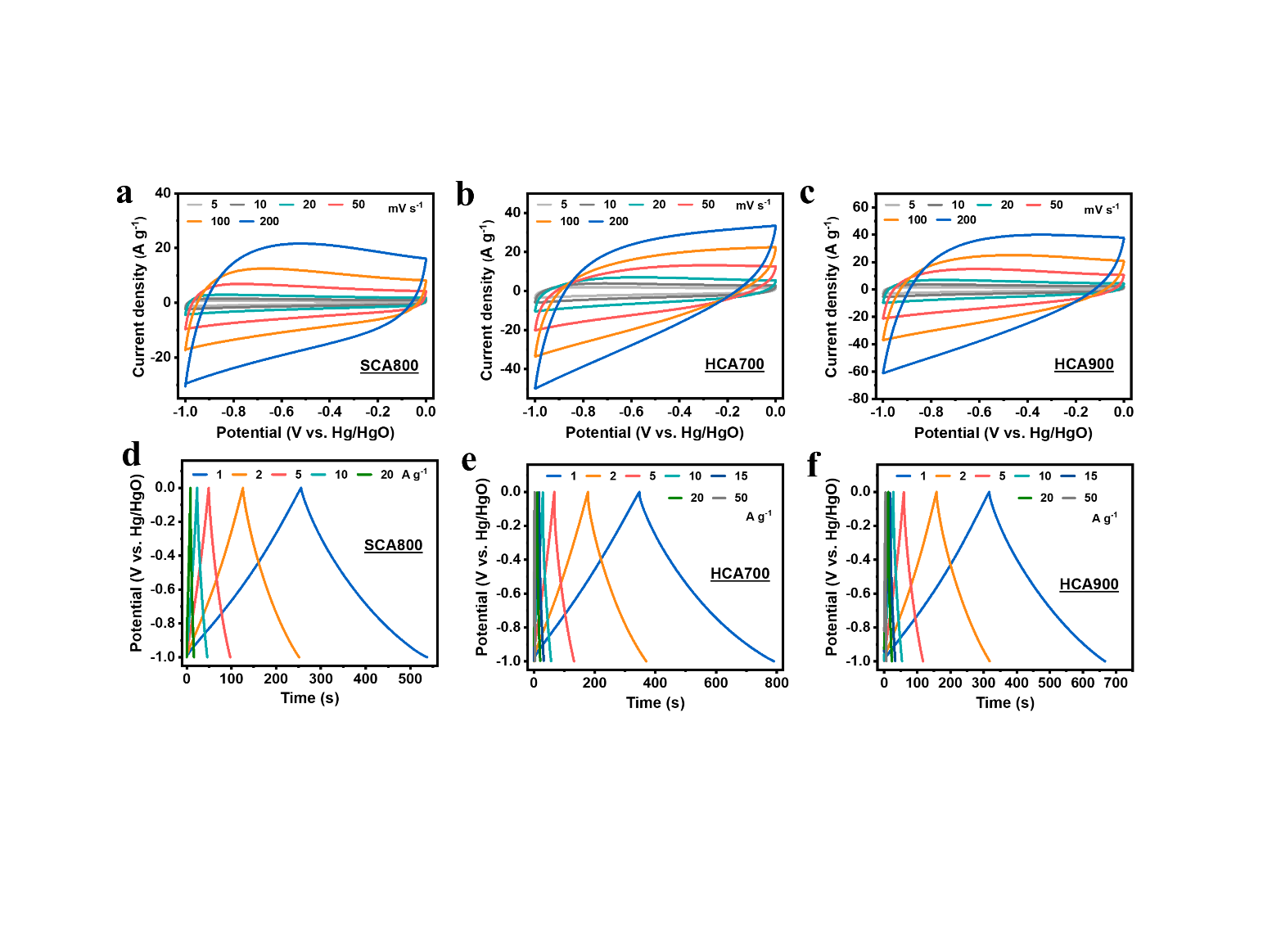


**Figure S21. Electrochemical performance of control samples.** (a, d) CV and GCD curves for SCA800. (b, e) CV and GCD curves for HCA700. (c, f) CV and GCD curves for HCA900. The distorted GCD profiles of HCA700 and the smaller CV area of SCA800, compared to HCA800 (main text), highlight the superior electrochemical kinetics and capacitance of the optimally synthesized material.


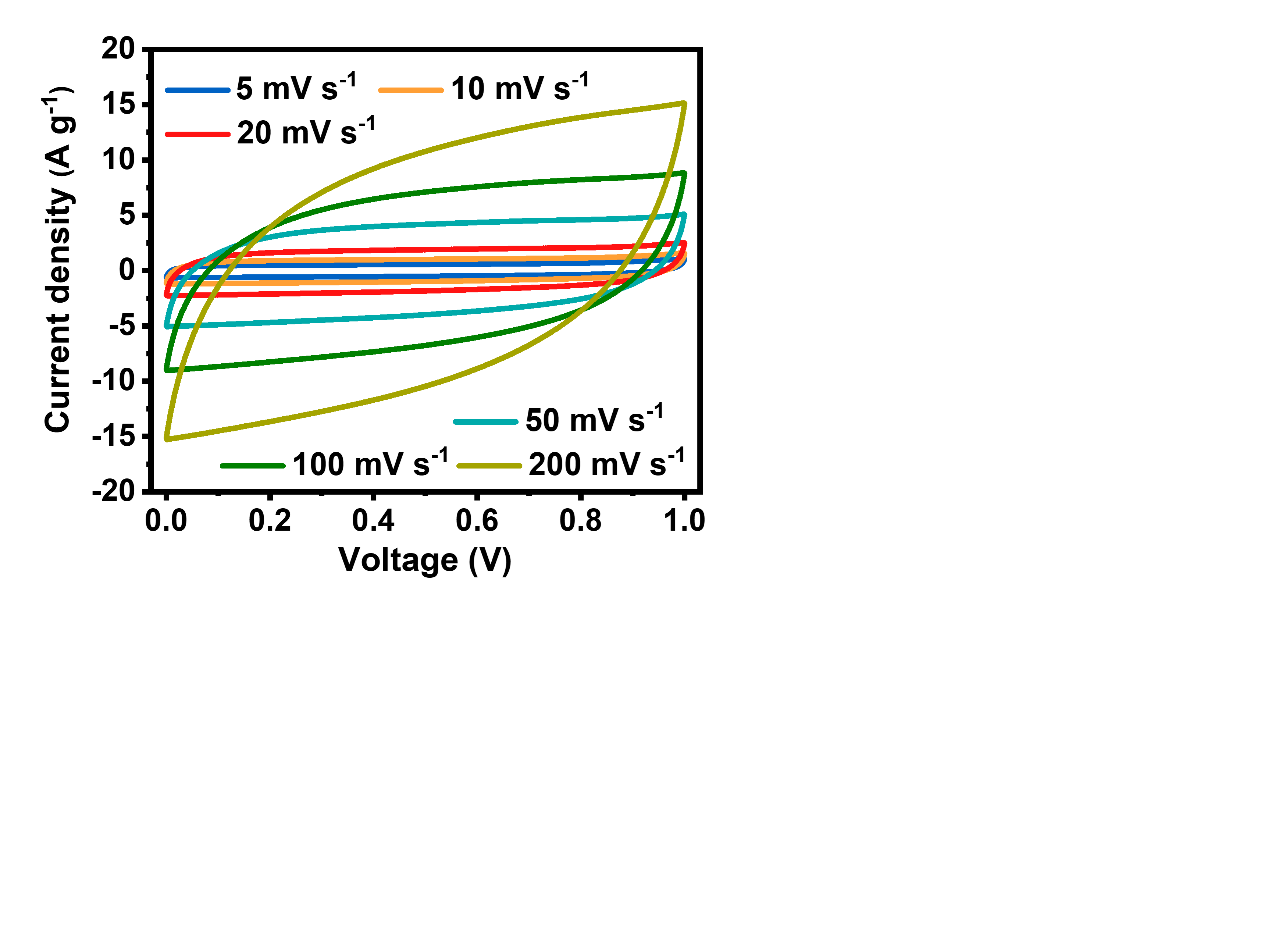


**Figure S22. CV curves of the HCA800-based symmetric supercapacitor at various scan rates from 5 to 200 mV s^-1^.** The device maintains a quasi-rectangular shape even at high scan rates, indicating excellent capacitive behavior and high-rate capability.


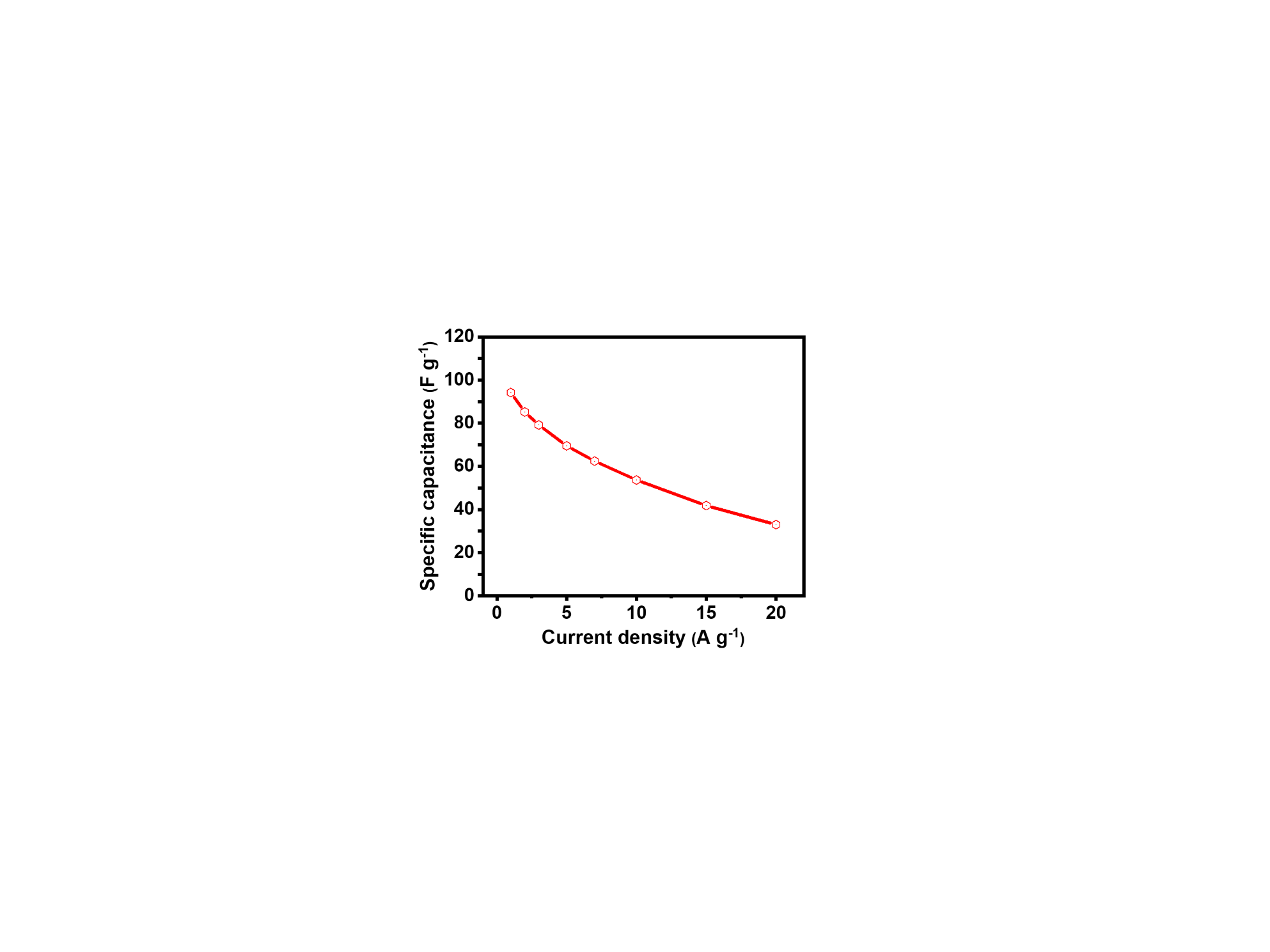


**Figure S23. Specific capacitance of the HCA800-based symmetric supercapacitor at different current densities.** The device delivers a specific capacitance of 94.2 F g^-1^ at 1 A g^-1^ and maintains good capacitance retention at higher rates.


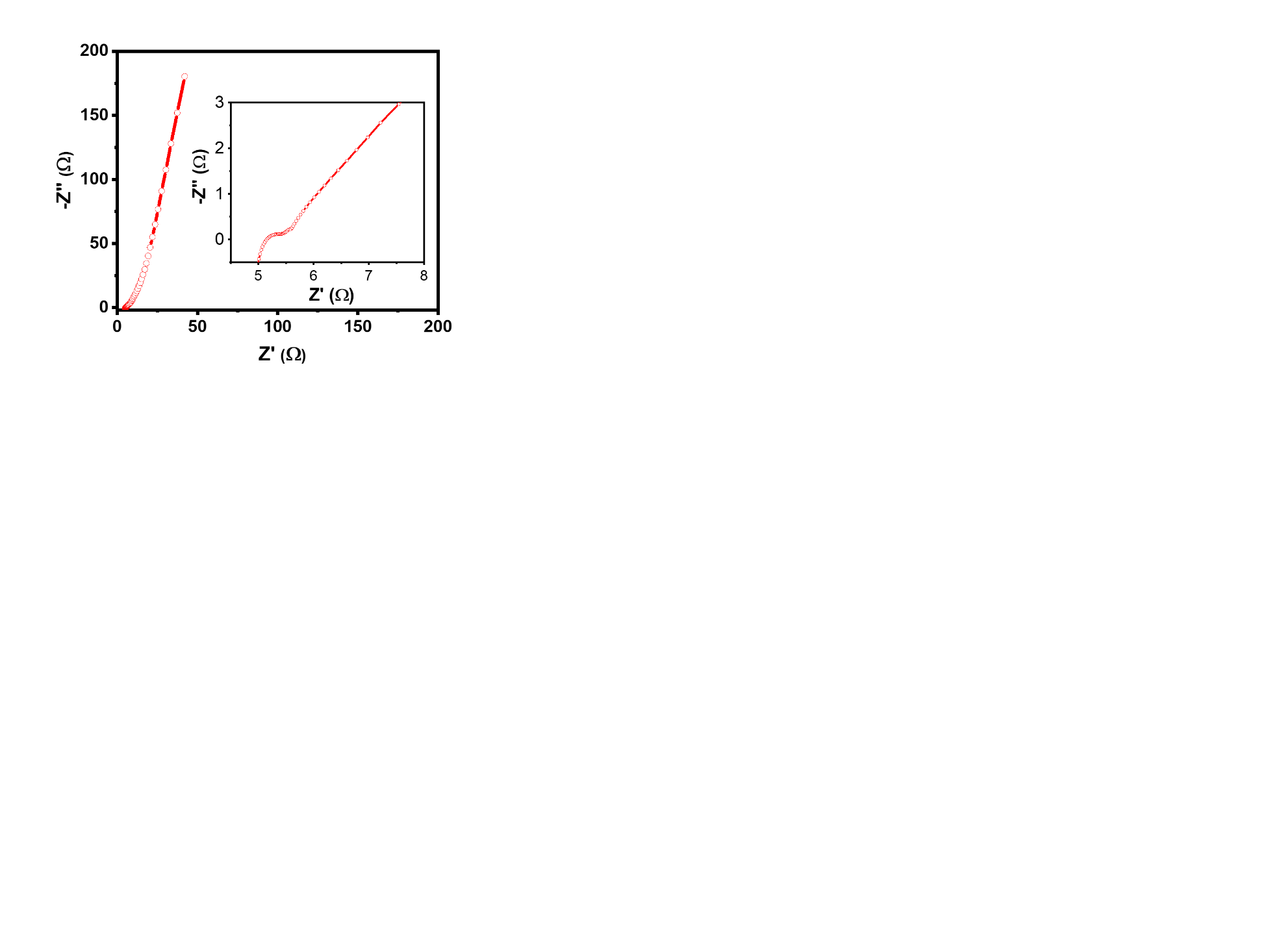


**Figure S24. Nyquist plot for the HCA800-based symmetric supercapacitor.** The small semicircle in the high-frequency region indicates a low charge-transfer resistance (Rct), while the nearly vertical line in the low-frequency region signifies efficient ion diffusion, collectively confirming rapid electrochemical kinetics within the device.


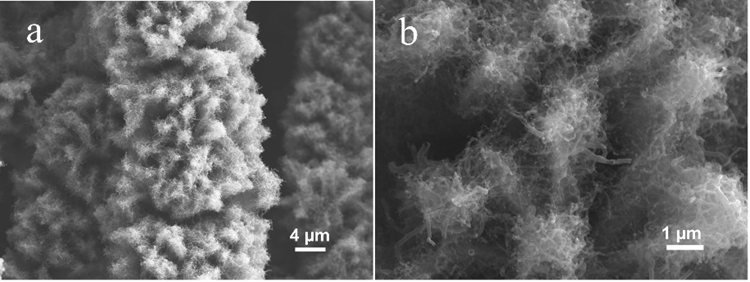


**Figure S25. Post-cycling SEM image of the HCA800 electrode after 10,000 charge-discharge cycles at 10 A g^-1^.** The hierarchical "array-on-array" architecture remains fully intact without any obvious structural degradation or detachment, providing direct evidence for the material's outstanding mechanical and electrochemical stability.


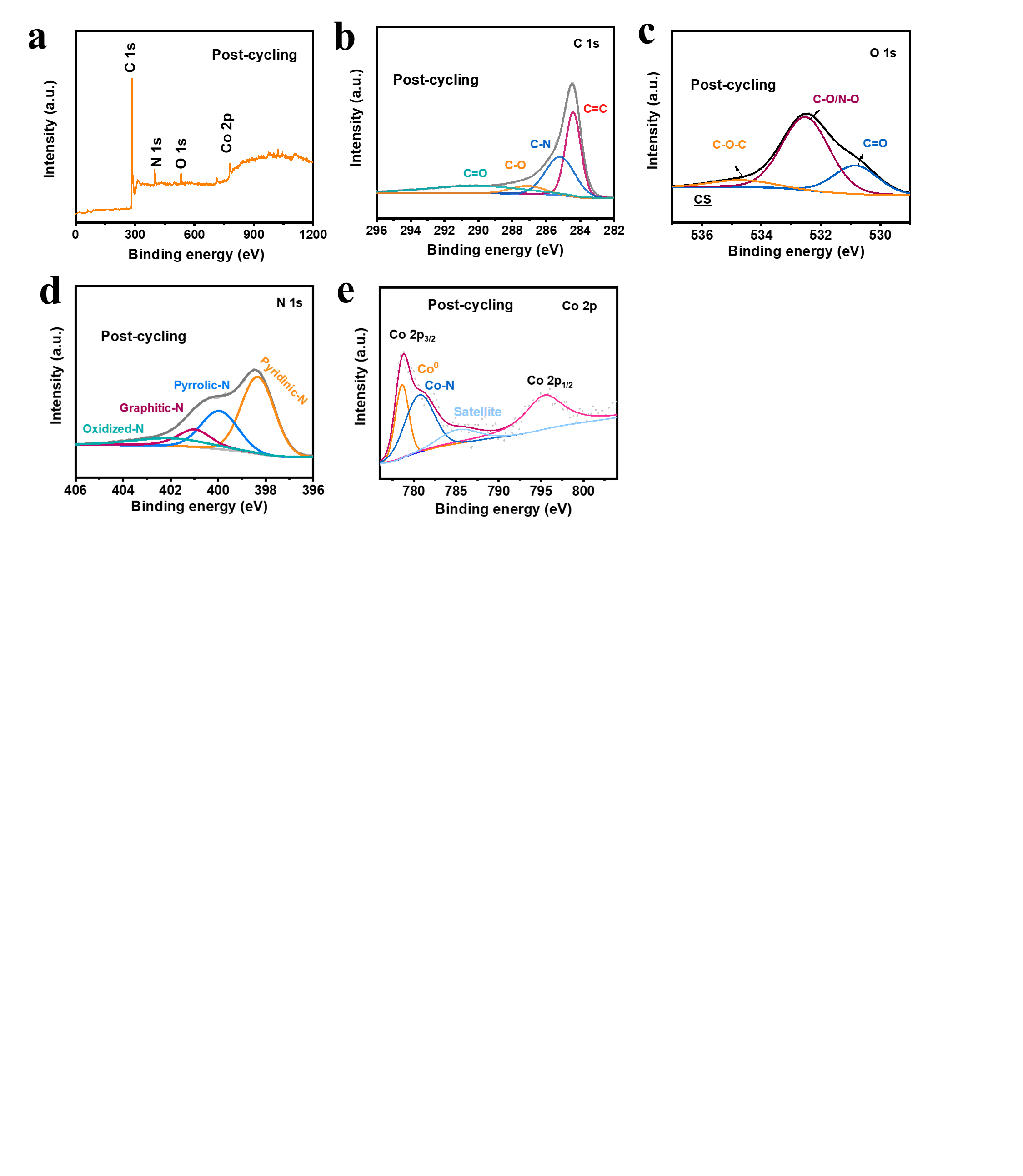


**Figure S26. Post-cycling XPS spectra of the HCA800 electrode after 10,000 charge-discharge cycles at 10 A g^-1^.** From the figure, it can be seen that after long-term cycling there is no significant change in either the elemental composition or the chemical states of the elements in the HCA800 electrode, indicating that the electrode exhibits not only stability of its multilevel structure but also excellent structural stability in terms of elemental composition.


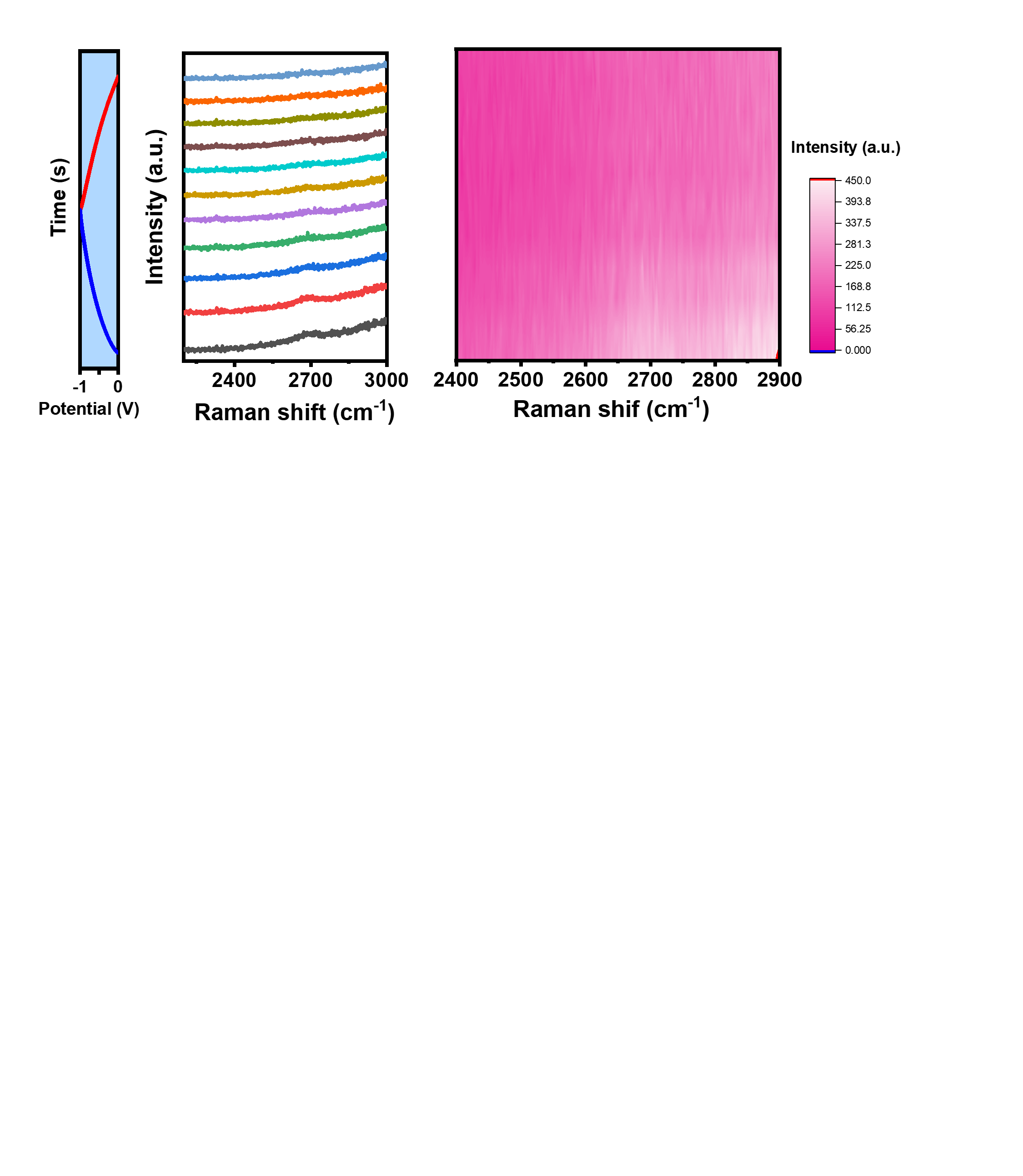


**Figure S27. GCD curve and *in situ* Raman spectra of the HCA800 electrode.** The 2D band in our system exhibits only subtle variations during charging/discharging, which are within the experimental noise and do not show a clear systematic shift or intensity trend. This is likely because the hierarchical carbon framework is highly defect-rich and structurally robust, causing changes in the 2D band to be less pronounced than in nearly ideal graphene.


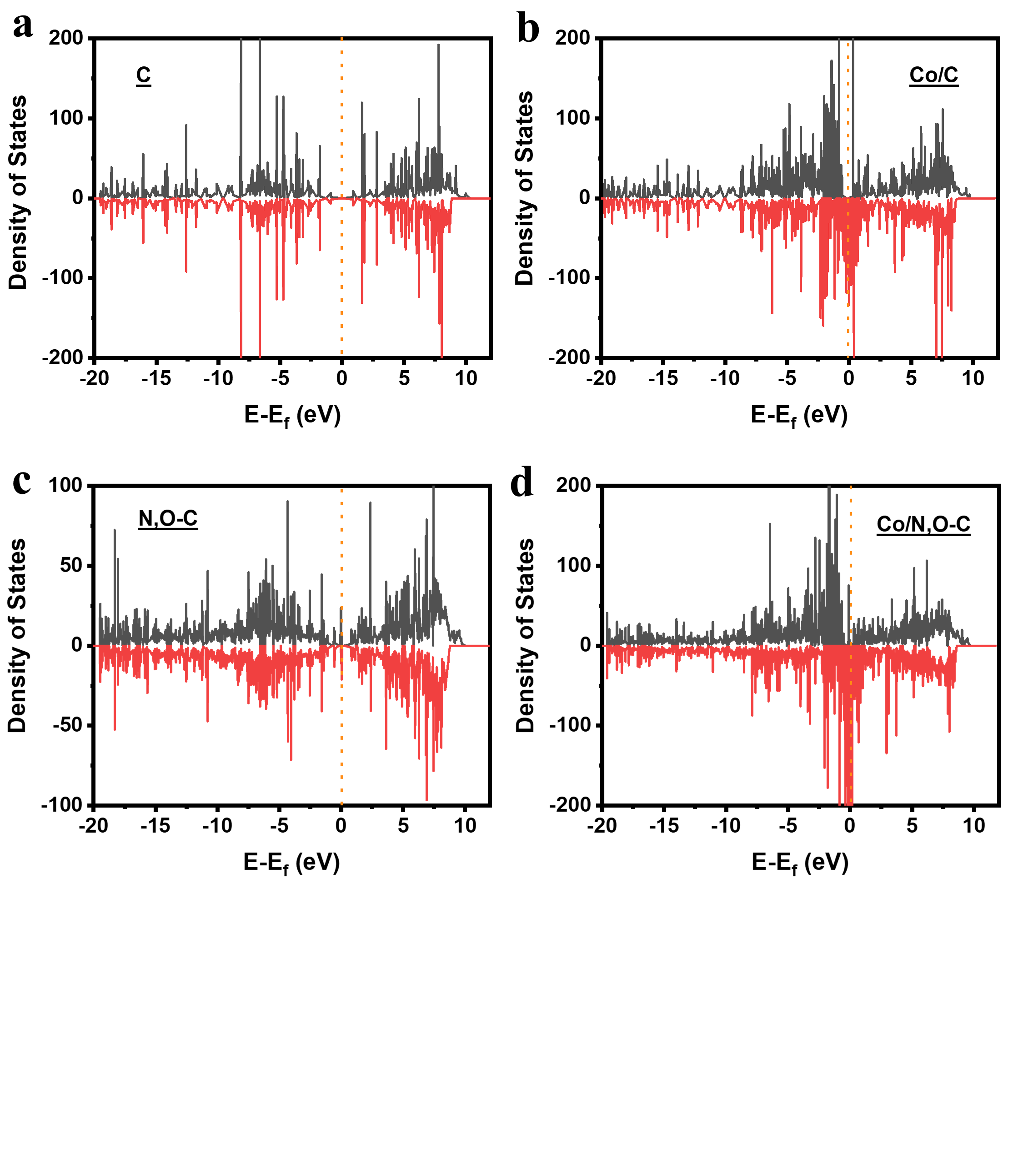


**Figure S28. Density of States plots for the (a) carbon matrix, Co/carbon, N,O-doped carbon, and Co/N,O-doped carbon models.**The density of states (DOS) calculations clarifies the electronic structure evolution. Carbon matrix exhibits a low DOS around the Fermi level, indicating limited charge carrier density. Upon anchoring Co onto the carbon matrix, pronounced Co–C hybridized states emerge around the Fermi level, evidencing enhanced electronic coupling and charge transfer. Introduction of N and O heteroatoms significantly increases the DOS near the Fermi level and generates abundant defect‑related states, which is beneficial for improving electronic transport and creating additional electrochemically active sites. Among all models, Co/N,O‑doped carbon displays the highest and most concentrated DOS near the Fermi level, together with a d‑band center shifted toward the Fermi level. This optimized electronic configuration facilitates fast electron hopping and reversible redox processes at Co centers and heteroatom sites, thus accounting for the superior rate capability and high specific capacitance of the Co/N,O‑C electrode.


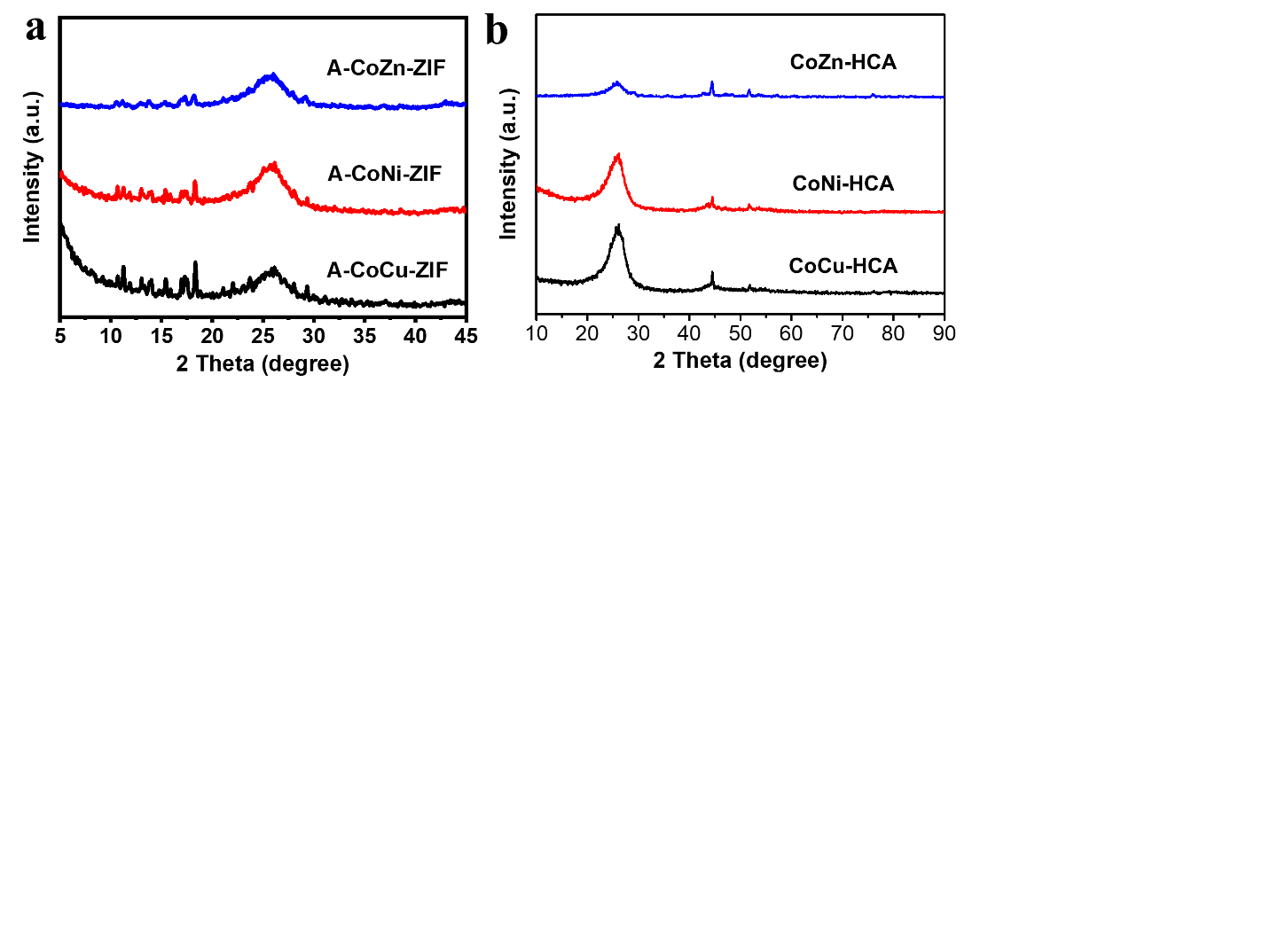


**Figure S29. XRD patterns of bimetallic precursors and their corresponding carbon derivatives.** (a) A-CoCu-ZIF, A-CoNi-ZIF, and A-CoZn-ZIF precursors. (b) CoCu-HCA, CoNi-HCA, and CoZn-HCA after vapor-assisted pyrolysis. The patterns confirm the successful conversion of the crystalline MOF precursors into graphitic carbon materials containing metallic alloy phases.


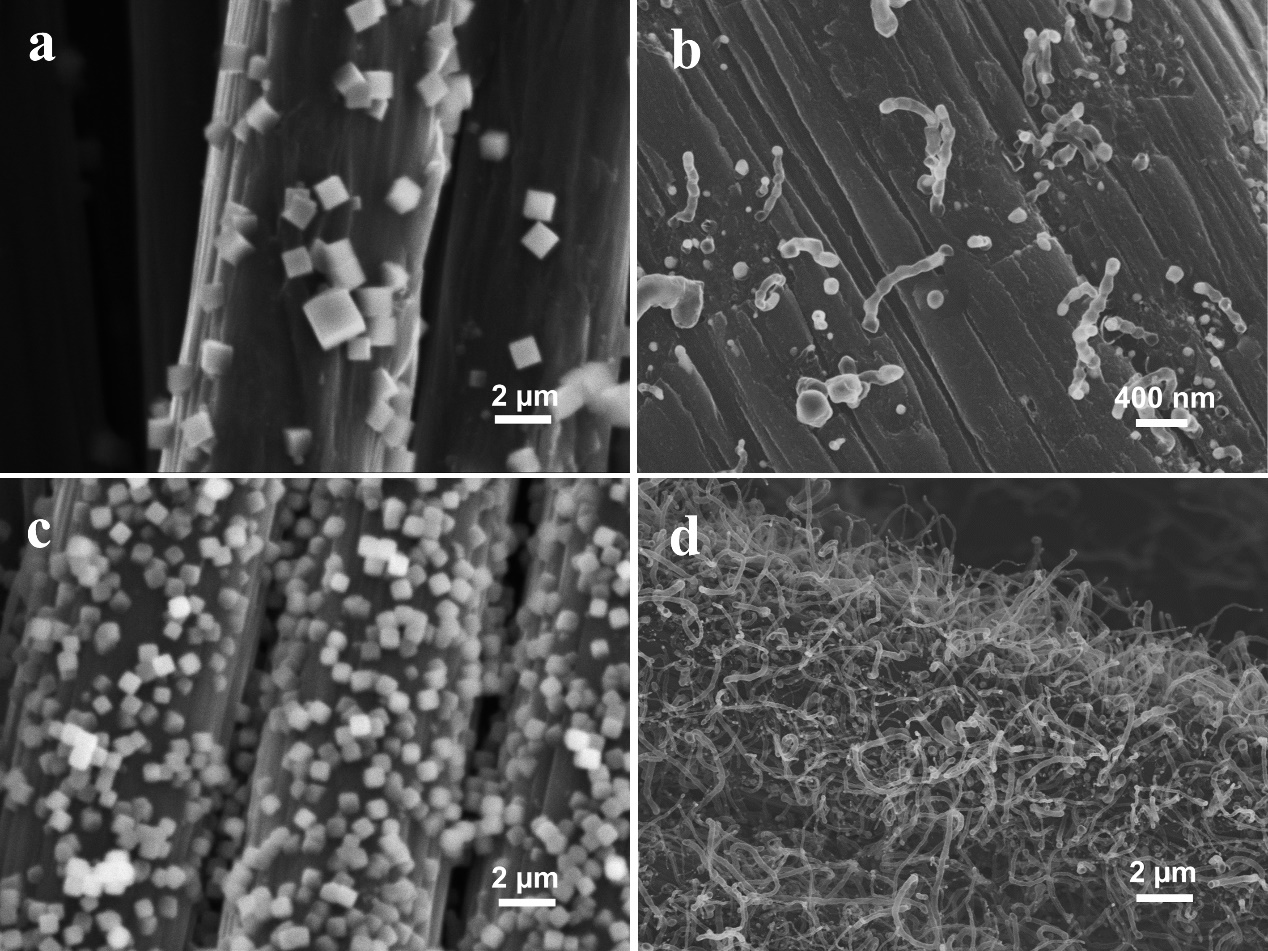


**Figure S30. SEM images demonstrating the generalizability of the vapor-assisted synthesis strategy to Prussian Blue Analogue (PBA) precursors.** (a) Co-Co-PBA precursor and (c) the resulting Co-CNT product. (b) Co-Ni-PBA precursor and (d) the resulting CoNi-CNT product. The successful transformation of PBA precursors into well-defined CNT architectures underscores the versatility of this remote regulation method.


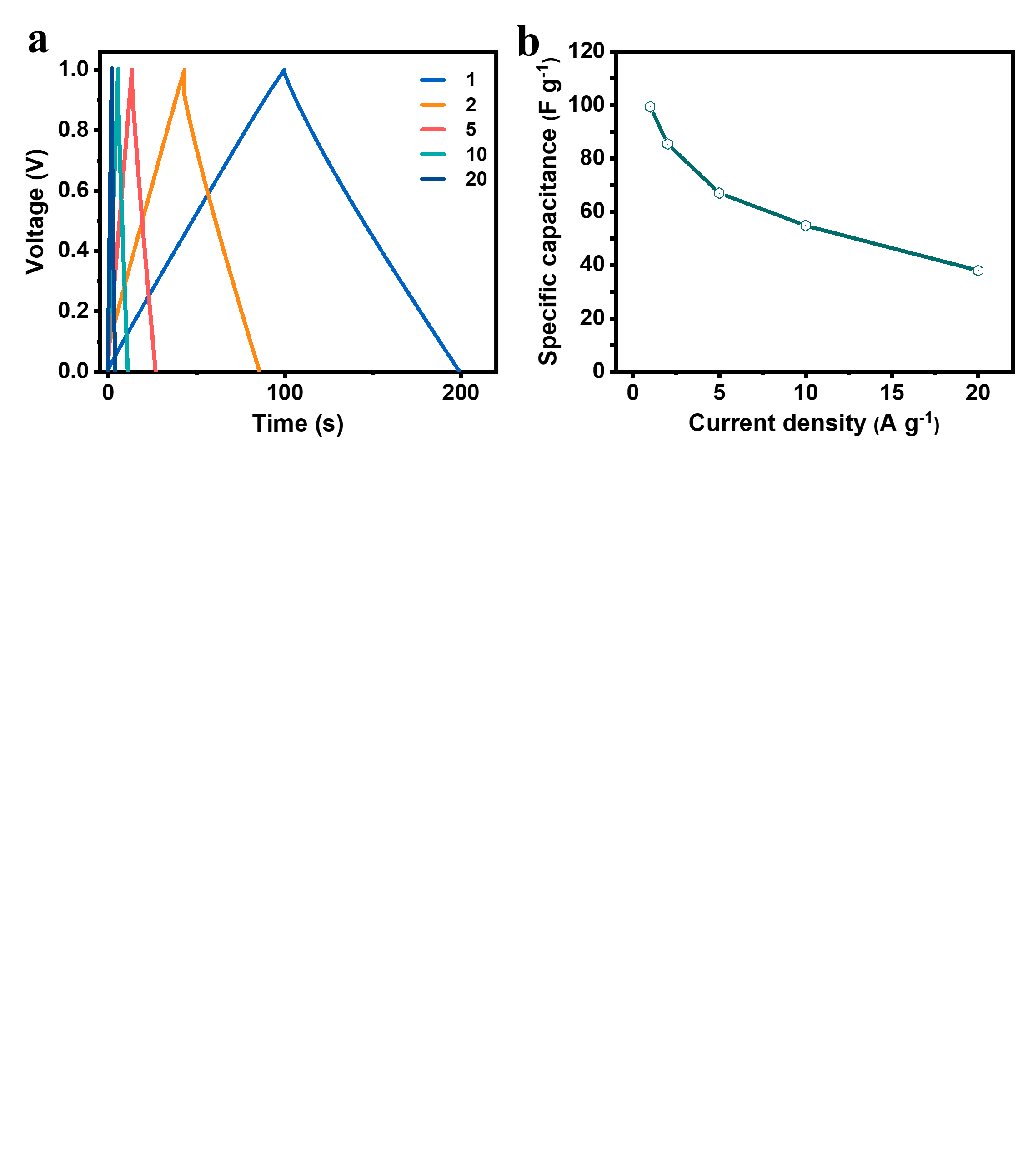


**Figure S31. (a) GCD curves and (b) Specific capacitance of the flexible symmetric supercapacitor.** Figure S31a shows the GCD curves of the HCA800-based flexible symmetric supercapacitor at various current densities. All curves display nearly isosceles triangular shapes with highly symmetric charge and discharge branches, indicating ideal capacitive behavior and excellent coulombic efficiency in the PVA/KOH gel electrolyte. As the current density increases, the discharge time decreases proportionally, confirming that the device can accommodate fast charge–discharge processes without significant polarization. Figure S31b presents the specific capacitance of the flexible symmetric supercapacitor as a function of current density. At low current density (e.g., 0.5–1 A g^-1^), the device delivers a relatively high specific capacitance, reflecting the effective utilization of the hierarchical carbon nanoarrays and accessible active sites. With increasing current density, the specific capacitance gradually decreases due to the shortened ion diffusion time and partial loss of deep pore accessibility, but the capacitance retention at high current density remains high. This behavior is consistent with the three-electrode results in the manuscript and demonstrates that the HCA800 electrode maintains fast ion/electron transport and robust rate capability even in the flexible solid-state configuration.


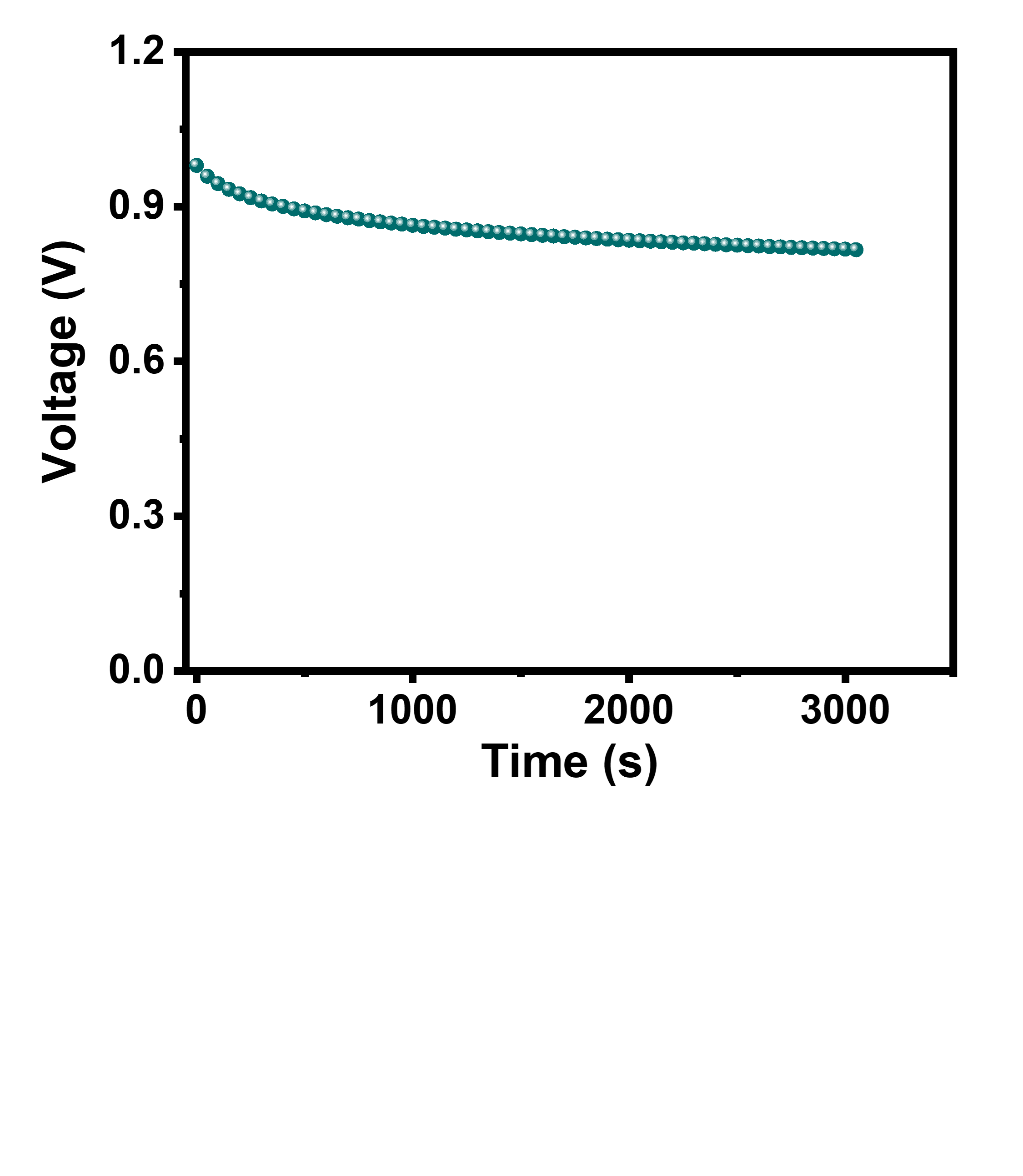


**Figure S32. Self-discharge behavior of the flexible symmetric supercapacitor.** In a typical measurement, the device is first charged to 1.0 V and then left at open-circuit conditions while the cell voltage is monitored as a function of time. The curves exhibit an initial relatively rapid voltage drop, which can be attributed to the redistribution of ions within the electric double layer and the relaxation of surface charges. After this transient stage, the voltage decay rate becomes significantly slower, indicating a low leakage current and the absence of severe redox side reactions at the electrode/electrolyte interface. Even after a prolonged rest period, the device retains a considerable fraction of its initial voltage, confirming that the flexible solid-state supercapacitor based on HCA800 possesses a low self-discharge rate and good charge-holding capability. This favorable self-discharge behavior is closely related to the well-ordered carbon framework, stable Co/N/O active sites, and the mechanically robust, conformal contact between the electrode and the gel electrolyte.


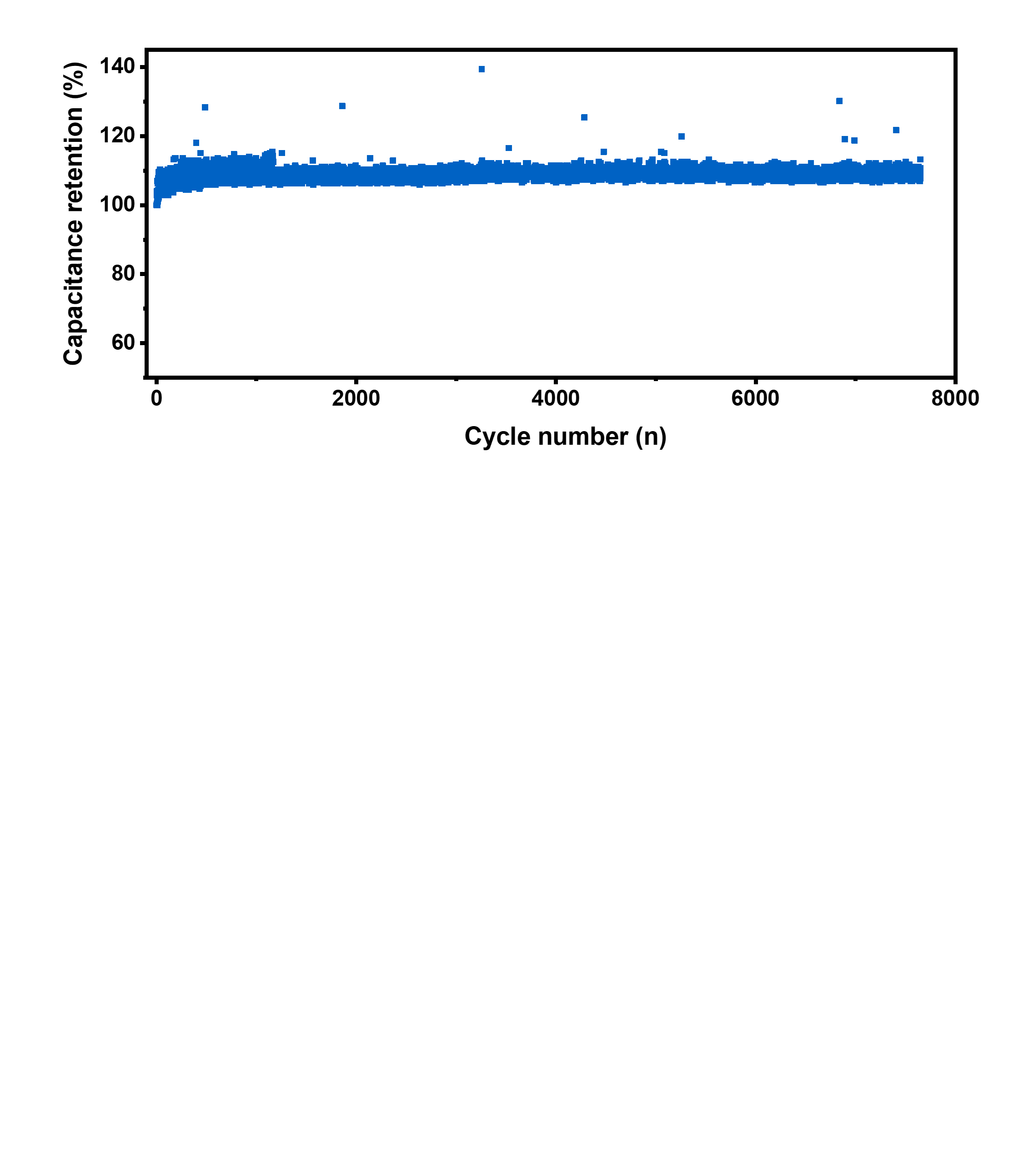


**Figure S33. Cycling stability of the flexible symmetric supercapacitor.** The device was subjected to continuous charge–discharge cycling at a relatively high current density of 10 A g^-1^ for a large number of cycles. The capacitance retention remains close to its initial value throughout the test, and the variation in capacitance with cycle number is smooth without obvious abrupt drops or progressive degradation segments. This result highlights the outstanding long-term electrochemical durability of the flexible device.


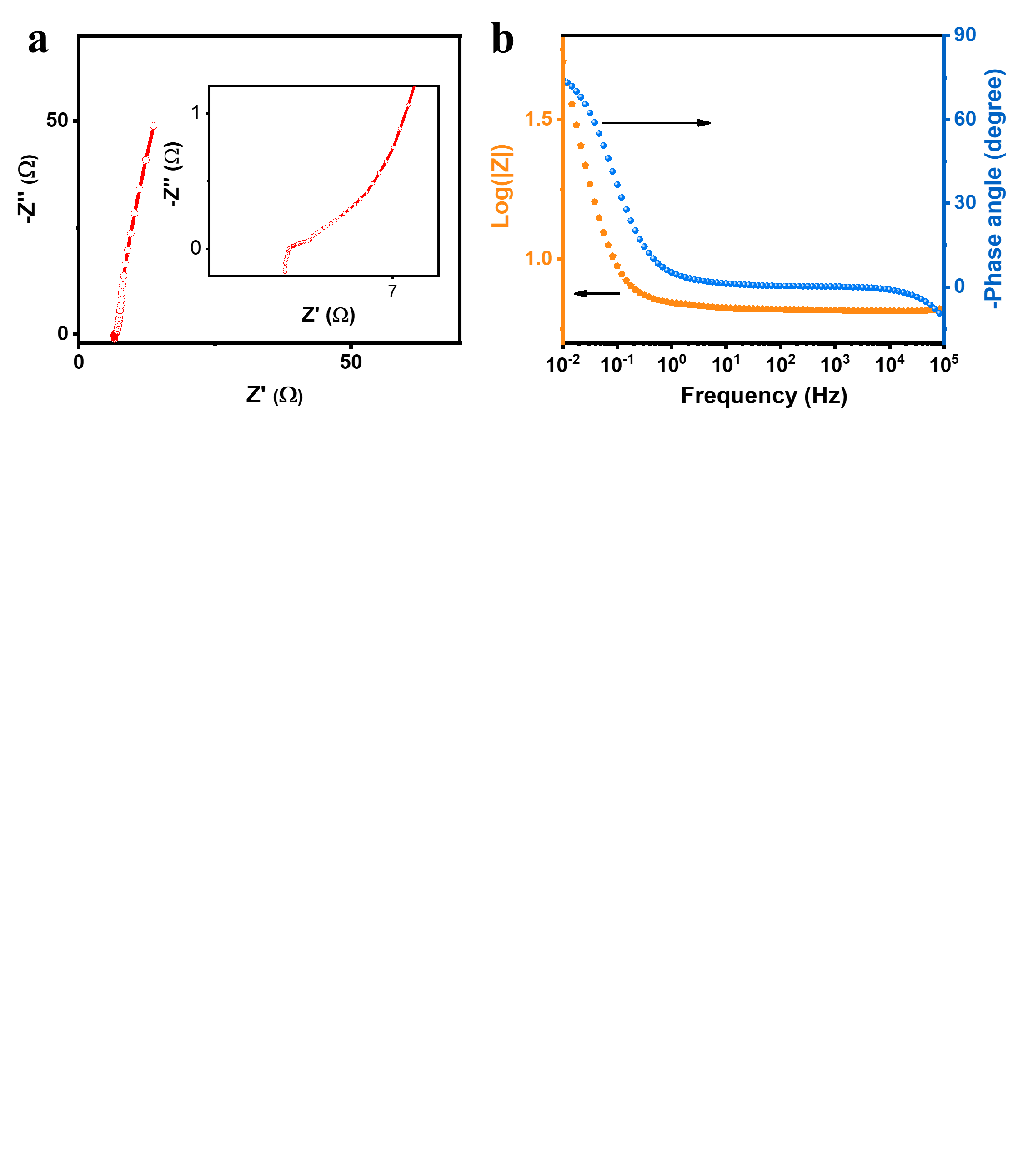


**Figure S34. Nyquist and Bode plots for the flexible symmetric supercapacitor.**  In the high-frequency region, the intercept of the curve on the real axis corresponds to the equivalent series resistance, which includes the intrinsic resistance of the current collectors, the resistance of the gel electrolyte, and the contact resistance at the electrode/electrolyte interfaces. The small R value indicates good ionic conductivity of the PVA/KOH gel and low ohmic losses within the device. The small semicircle observed at high to intermediate frequencies is associated with the charge-transfer resistance at the electrode–electrolyte interface; its small diameter reveals that charge transfer is highly efficient on the HCA800 surface. In the low-frequency region, the Nyquist plot approaches a nearly vertical line, characteristic of ideal capacitive behavior and efficient ion diffusion within the hierarchical pore structure. This impedance response is in good agreement with that of the aqueous symmetric cell (Figure S24), confirming that the transition to a flexible solid-state configuration does not compromise the intrinsic electrochemical kinetics. Figure S34b presents the corresponding Bode plots, including the magnitude of impedance and the phase angle as a function of frequency. At low frequency, the phase angle approaches −80°, indicating that the device behaves close to an ideal capacitor.


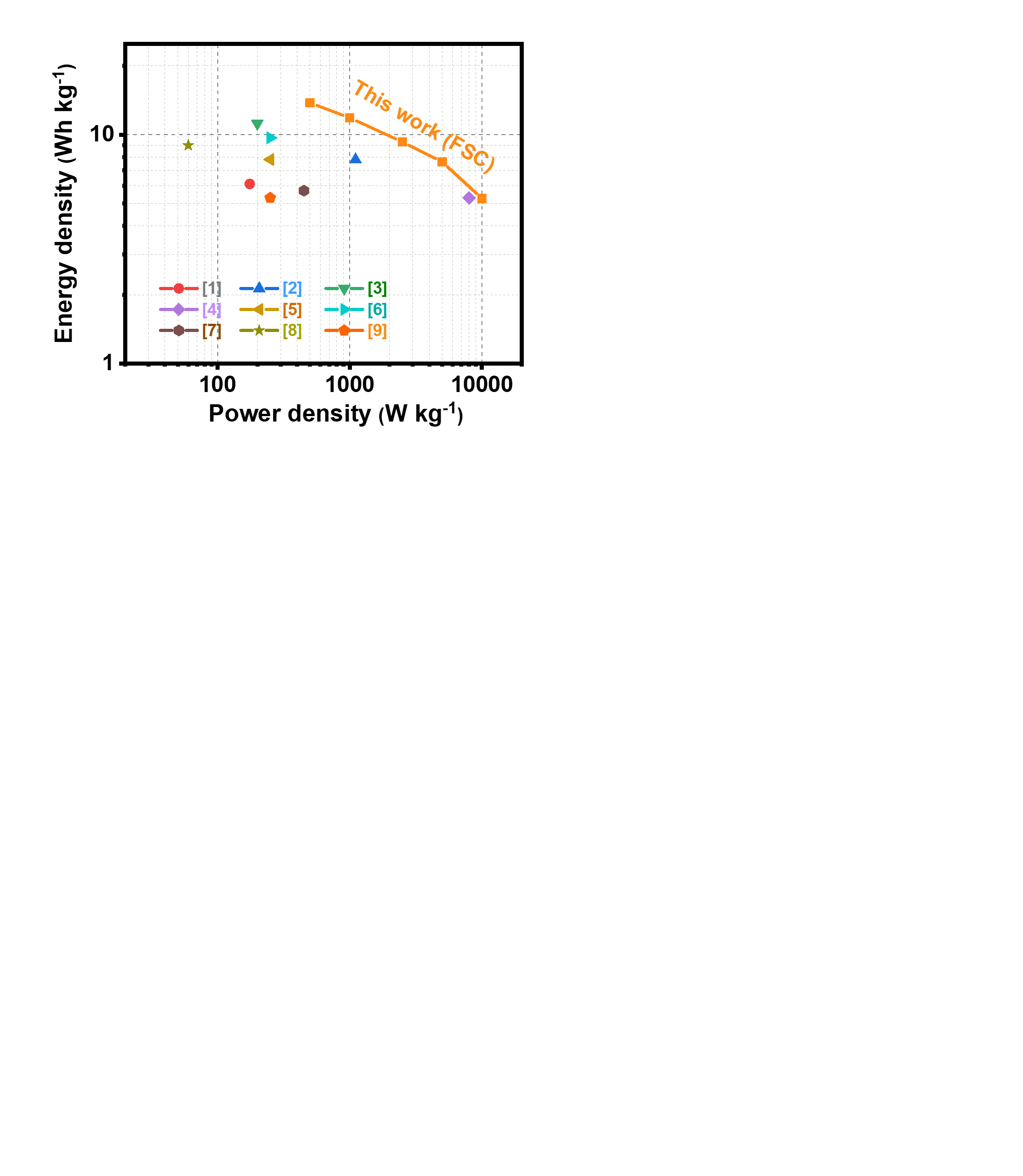


**Figure S35. Ragone plot of the flexible symmetric supercapacitor with recently reported carbon-based flexible supercapacitor.** ^[1-9]^ The device delivers a high energy density of 13.81 Wh kg^-1^, which is comparable to the aqueous symmetric configuration discussed in the main text (Figure 4h). As the power density increases to 10 kW kg^-1^, the energy density gradually decreases, as expected for capacitive systems, yet remains at a competitive level, demonstrating that the flexible solid-state device can simultaneously provide high power output and appreciable energy storage. The favorable position of the HCA800-based flexible supercapacitor in the Ragone plot, especially when benchmarked against recently reported carbon-based flexible supercapacitors, highlights the advantages of the vapor-assisted constructed hierarchical carbon nanoarrays. The combination of high specific surface area, interconnected CNT network, and optimized Co/N/O surface chemistry enables rapid charge transport and efficient ion accessibility, thereby affording an excellent balance between energy and power densities in a mechanically robust and bendable device format. This confirms the strong potential of the HCA800 electrodes for practical, wearable, and portable energy storage applications.

6. Supporting Tables

**Table S1. Elemental composition of carbon samples determined by XPS.**

|  | C (at.%) | N (at. %) | O (at. %) | Co (at. %) |
| --- | --- | --- | --- | --- |
| HCA700 | 83.65 | 9.74 | 5.00 | 1.61 |
| HCA800 | 84.85 | 9.60 | 4.05 | 1.50 |
| HCA900 | 90.01 | 6.24 | 1.93 | 1.82 |
| SCA800 | 86.52 | 8.78 | 3.39 | 1.31 |

**Table S2. Comparison of the capacitive performance of the HCA800 electrode with other reported porous carbon materials in a three-electrode system.**

| Porous carbon | Current Density (A g^-1^) / Scan Rate (mV s^-1^) | Specific Capacitance (F g^-1^) | Self-standing | Reference |
| --- | --- | --- | --- | --- |
| HCA800 | 1 / 50 | 360 / 191 | Yes | This work |
| NHPC-60 | 0.5 / 100 | 219 / 120 | No | ^[10]^ |
| CK_1_K_2_-122 | 1 | 296 | No | ^[11]^ |
| AC-800-2 | 0.05 /5 | 324 / 286 | No | ^[12]^ |
| APNC | 1 / 30 | 360 / 157 | No | ^[13]^ |
| CF2CT | 0.5 / 20 | 365 / 286 | No | ^[14]^ |
| CA-10 | 0.5 / 25 | 323 / 225 | No | ^[15]^ |
| AC-O-B | 0.5/10 | 214 / 133 | No | ^[16]^ |
| NPC−2 | 0.25 / 50 | 234 / 143 | No | ^[17]^ |
| HPC-5 | 0.5 / 50 | 359 / 225 | No | ^[18]^ |
| WPU800 | 1 | 241 | No | ^[19]^ |
| WR-600 | 10 / 200 | 248 / 160 | No | ^[20]^ |
| HLPC | 0.2 / 20 | 380 / 244 | No | ^[21]^ |

**Table S3. Comparison of the capacitive performance of the HCA800 electrode with other recently reported MOF-derived carbon materials.**

| Pristine MOF | Potential window (V) | Current Density (A g^-1^) / Scan Rate (mV s^-1^) | Capacitance  (F g^-1^) | Self-standing | Reference |
| --- | --- | --- | --- | --- | --- |
| Co-ZIF | -1.0-0 | 1 / 50 | 360 / 191 | Yes | This work |
| ZIF-8 | -1.0-0 | 0.2 / 200 | 269 / 140 | No | ^[22]^ |
| ZIF-8 | 0-0.8 | 1 / 10 | 206 / 166 | No | ^[23]^ |
| MOF-5 | 0-2.5 | 1 / 50 | 200 / 140 | No | ^[24]^ |
| ZIF-8 | -1.0-0 | 1 / 10 | 272 / 196 | No | ^[25]^ |
| Zn-MET | 1.0-0 | 0.2 / 20 | 361 / 157 | No | ^[26]^ |
| ZIF-8 | 0-1.0 | 0.5 / 15 | 259 / 143 | No | ^[27]^ |

**Reference**

[1] L. H. Xu, Y. T. He, Y. Xu, S. Sun, J. Liu, J. Yang, J. L. Wen, T. Q. Yuan, *Adv. Funct. Mater.* **2025**, 35, 2501263;

[2] Y. Wang, M. Q. Chen, T. J. Liu, Y. Q. Cao, Y. F. Liu, *ACS Appl. Energy Mater.* **2025**, 8, 12627;

[31] Z. Shen, D. Jia, W. Zhou, K. Zheng, H. Li, Y. Sang, Y. Lv, J. Qiu, X. He, *Energy Environ. Mater.* **2025**, DOI: 10.1002/eem2.70135;

[4] S. Sarsenov, R. A. Senthil, C. J. Moon, A. Kumar, V. Maheskumar, M. Ubaidullah, M. Y. Choi, J. Mater. Chem. A **2025**, DOI: 10.1039/d5ta06392b;

[5] Z. Ren, Y. Pei, X. Wu, Y. Lv, R. Xue, J. Guo, X. Zhang, *Energy & Environmental Materials* **2025**, DOI: 10.1002/eem2.70161;

[6] X. H. Jiao, H. L. Liu, X. F. Wei, B. C. Yang, B. B. Chang, *Acs Applied Energy Materials* **2025**, DOI: 10.1021/acsaem.5c02233;

[7] I. L. Egun, B. Akinwolemiwa, H. Y. He, M. C. Ma, Z. F. Chen, G. Z. Chen, D. Hu, *Chem. Eng. J.* **2025**, 509, 161386;

[8] H. Wang, Y. Yuan, F. Q. Xiong, B. L. Ma, J. M. Yang, Y. Qing, F. X. Chu, Y. Q. Wu, *Chem. Eng. J.* **2023**, 476, 146640;

[9] H. Liang, R. Shi, Y. Zhou, W. Jiang, T. Sun, Z. Zhang, L. Sun, J. Lian, H. Li, Y. Bu, *ACS Energy Lett.* **2022**, 7, 4381.

[10] Y. L. Zhu, T. Wang, Y. Ma, R. Sheng, P. X. Ren, J. Guo, D. Z. Jia, D. L. Wu, *Chem. Eng. J.* **2025**, 513, 162989.

[11] Y. Zhang, X. Xu, Q. Geng, Q. Li, X. Li, Y. Wang, Z. Tang, B. Gao, X. Zhang, P. K. Chu, K. Huo, *Chem. Science* **2025**, 16, 2034.

[12] Y. Wang, M. Q. Chen, T. J. Liu, Y. Q. Cao, Y. F. Liu, *ACS Appl. Energy Mater.* **2025**, 8, 12627.

[13] S. Sarsenov, R. A. Senthil, C. J. Moon, A. Kumar, V. Maheskumar, M. Ubaidullah, M. Y. Choi, *J. Mater. Chem. A* **2025**, DOI: 10.1039/d5ta06392b.

[14] Z. Ren, Y. Pei, X. Wu, Y. Lv, R. Xue, J. Guo, X. Zhang, *Energy Environ. Mater.* **2025**, DOI: 10.1002/eem2.70161.

[15] Y. Y. Peng, M. M. Yu, L. Zhao, H. Z. Zeng, C. H. Zhao, G. Y. Li, J. Zhang, G. Liu, C. A. Etogo, Y. Z. Wu, F. Ran, *Energy Storage Mater.* **2025**, 75, 104080.

[16] Z. Li, Z. Li, C. Di, D. Zhang, Q. Wang, H. Sun, Q. Sun, F. Yuan, R. Li, B. Wang, *Carbon* **2025**, 244, 120648.

[17] Y. Li, L. Yang, R. Xiong, Y. Shang, Y. Tian, B. Chi, B. Han, K. Xia, H. Wang, Q. Wang, *Small* **2025**, 21, 2500421.

[18] X. Jiao, H. Liu, X. Wei, B. Yang, B. Chang, *ACS Appl. Energy Mater.* **2025**, DOI: 10.1021/acsaem.5c02233.

[19] X. R. Huang, Y. J. Cao, J. B. Xu, Z. T. Bian, X. X. Zhao, Y. Y. Qin, L. Y. Fang, Y. Tang, T. T. Cui, K. Y. Zhang, *J. Power Sources* **2025**, 653, 237761.

[20] I. L. Egun, B. Akinwolemiwa, H. Y. He, M. C. Ma, Z. F. Chen, G. Z. Chen, D. Hu, *Chem. Eng. J.* **2025**, 509, 161386.

[21] H. Wang, Y. Yuan, F. Q. Xiong, B. L. Ma, J. M. Yang, Y. Qing, F. X. Chu, Y. Q. Wu, *Chem. Eng. J.* **2023**, 476, 146640.

[22] Z. Shen, D. Jia, W. Zhou, K. Zheng, H. Li, Y. Sang, Y. Lv, J. Qiu, X. He, *Energy Environ. Mater.* **2025**, DOI: 10.1002/eem2.70135.

[23] D. Kim, J. Park, S. Jung, J. Jang, M. Han, M. Kim, W. K. Zhu, W. J. Song, Y. Yamauchi, J. Kim, *Nanoscale* **2025**, 17, 10344.

[24] T. T. Tang, R. L. Yuan, N. N. Guo, J. Y. Zhu, X. M. Gan, Q. Q. Li, F. W. Qin, W. X. Luo, L. X. Wang, S. Zhang, H. H. Song, D. Z. Jia, *J. Colloid. Interf. Sci.* **2022**, 623, 77.

[25] C. Ma, Y. H. Mo, L. Liu, Y. F. Yu, A. B. Chen, *Chinese Chem. Lett.* **2021**, 32, 1485.

[26] D. D. Jia, Z. L. Shen, W. Zhou, Y. Li, J. He, L. Jiang, Y. C. Wei, X. J. He, *Chem. Eng. J.* **2024**, 485, 149820.

[27] D. L. Zhang, J. H. Zhang, M. D. Pan, Y. Wang, T. Sun, *J. Alloy. Compd.* **2021**, 853, 157368.
